# AAV-BDNF modulates 5HT2A receptor, glycine receptor subunits, and Nav1.1 channel gene expression in lumbar motoneurons after spinal cord injury

**DOI:** 10.1007/s11033-026-12984-4

**Published:** 2026-11-01

**Authors:** Benjun Ji, Anna Głowacka, Julita Czarkowska-Bauch, Olga Gajewska-Woźniak, Małgorzata Skup

**Affiliations:** 1 Nencki Institute of Experimental Biology Polish Academy of Sciences, 3 Pasteur Street, Warsaw, 02-093 Poland; 2 Key Laboratory of Spine and Spinal Cord Injury Repair and Regeneration of Ministry of Education, School of Life Sciences and Technology, Tongji University, Shanghai, 200065 China; 3 Mossakowski Medical Research Institute, Neurotoxicology Department, Polish Academy of Sciences, 5 Adolfa Pawińskiego Street, Warsaw, 02-106 Poland

**Keywords:** Spinal cord transection, BDNF overexpression, TrkB upregulation, Neurotransmitter receptors, Ion channels, Lumbar motoneuron

## Abstract

**Background:**

Proper excitability of spinal motor neurons (MNs) is essential for motor output. Spinal cord injury (SCI) disrupts this network and compromises MN- driven skeletal muscle contractions. We previously showed that tracer-identified MNs innervating ankle extensor and flexor muscles display gene-expression changes toward a more excitatory profile at 2 weeks post-lesion in rats, however, this shift does not restore stepping. Given evidence that brain-derived neurotrophic factor (BDNF) enhances MN excitability and promotes functional recovery, we investigated its effects on gene expression following SCI.

**Methods:**

Adult male rats received retrograde tracers in gastrocnemius lateralis (GL) and tibialis anterior (TA) muscles. Two weeks later, complete low-thoracic transection was followed by bilateral lumbar L1-L2 injection of AAV-BDNF or PBS. Locomotion was assessed on a treadmill and in an open field. BDNF and TrkB transcripts were assessed by RNAscope, while selected receptor, transporter, and ion-channel transcripts were measured in lumbar segments and laser-microdissected GL and TA MNs by RT-qPCR.

**Results:**

At two weeks, AAV-BDNF increased spinal BDNF transcript levels and restored TrkB transcripts in lumbar MNs. It improved treadmill locomotion with plantar placement, alternating step-like movements, and body-weight support, while open-field hindlimb movements were also improved. BDNF selectively increased Htr2a transcripts, particularly in GL MNs, and reduced glycine receptor subunit transcripts. It also reversed SCT-induced Scn1a upregulation in GL and TA MNs. Changes in glutamatergic and GABAA receptor transcripts were selective and MN-population dependent.

**Conclusions:**

These findings show that intraspinal AAV-BDNF is associated with locomotor recovery and transcriptional changes in MN excitability-related genes. The results support a role for BDNF in spinal circuit reactivation after SCT.

**Supplementary Information:**

The online version contains supplementary material available at https://doi.org/10.1007/s11033-026-12984-4.

## Introduction

The most debilitating consequence of severe spinal cord injury (SCI) is the loss of motor function leading to paraplegia or tetraplegia depending on the level of the lesion [1–3]. Following SCI, the disruption of descending supraspinal input combined with local circuit reorganization, profoundly alters the excitability of motoneurons (MNs) - an intrinsic neuronal property determined by the density and function of membrane neurotransmitter receptors and ion channels [4–7]. We have previously demonstrated that, in the subacute phase (two weeks) following complete spinal cord transection (SCT) at the lower thoracic level, lumbar MNs exhibit a shift in the expression of genes encoding membrane proteins involved in excitatory and inhibitory neurotransmission toward a more excitatory profile. However, this intrinsic adaptation is insufficient to restore the stepping function [8].

One promising therapeutic strategy for SCI involves delivery of the brain derived neurotrophic factor (BDNF) transgene to the injured spinal cord or targeting its high affinity receptor TrkB to MNs [9–17]. In a previous study in rats, we demonstrated that BDNF overexpression caudal to the site of SCT promoted hindlimb stepping with body weight support during the subacute post-lesion phase. These findings suggested that BDNF overexpression facilitates circuit plasticity and neurotransmission by amplifying certain MN inputs but the molecular basis of this enhanced MN responsiveness remained elusive. We and others have shown that BDNF can directly modulate the properties of MNs [11, 18, 19]. BDNF overexpression upregulated the cholinergic phenotype in MNs, as evidenced by increased transcripts for the vesicular acetylcholine transporter (VAChT) and acetylcholinesterase (AChE) in soleus (Sol) MNs. Furthermore, BDNF partially preserved neuromuscular junctions in the Sol muscle during the subacute post-lesion phase [20].

Increasing evidence identifies BDNF/TrkB signaling as a key modulator of MN excitability across multiple levels of organization. It enhances monosynaptic EPSPs via postsynaptic NMDA receptors in neonatal MNs and increases NMDA receptor expression after spinal hemisection in adults [21–23]. BDNF applied to the medial gastrocnemius (MG) muscle to increase its concentration in MG innervating MNs enhances their electrical excitability [19]. Similarly, intraspinal administration of AAV-BDNF increased the excitability of ankle extensor MNs, as indicated by a reduced rheobase, in completely transected spinal cords [24]. These effects were proposed to result from enhanced excitatory synaptic transmission [25, 26]. However, a comprehensive understanding of the spectrum of molecular changes in MNs following BDNF enrichment and their relationship to locomotor recovery remains lacking.

In this study, we address two key questions: (1) does BDNF overexpressed in the lumbar spinal cord regulate the expression of its high affinity receptor TrkB in MNs, thereby modulating BDNF signalling and (2) how does BDNF overexpression alter the expression of genes encoding receptors and channels that contribute to the excitability of the lumbar neuronal circuit, specifically in extensor and flexor MNs innervating the ankle muscles. To address these questions, SCT at the thoracic Th11 level [8, 11, 26–28] was followed by bilateral injection of AAV-BDNF (SCT-BDNF rats) or PBS (SCT-PBS rats) into the lumbar L1 segment. Given that BDNF treatment promotes locomotion recovery in part by modulating MNs excitability, we focused our analysis on the second and third weeks post-injury, a period during which clear locomotor improvements have been observed [11, 24]. We show for the first time that intraspinal AAV-BDNF delivery restores TrkB transcript levels and selectively modulates genes encoding the serotonin 5-HT2A receptor, glycine receptor, and Nav1.1 channels in MNs. These changes in key regulators of MN excitability are associated with reactivation of spinal locomotor circuits and improved locomotor performance on treadmill and open-field tests.

## Materials and methods

### Animals

**Fig. 1:**
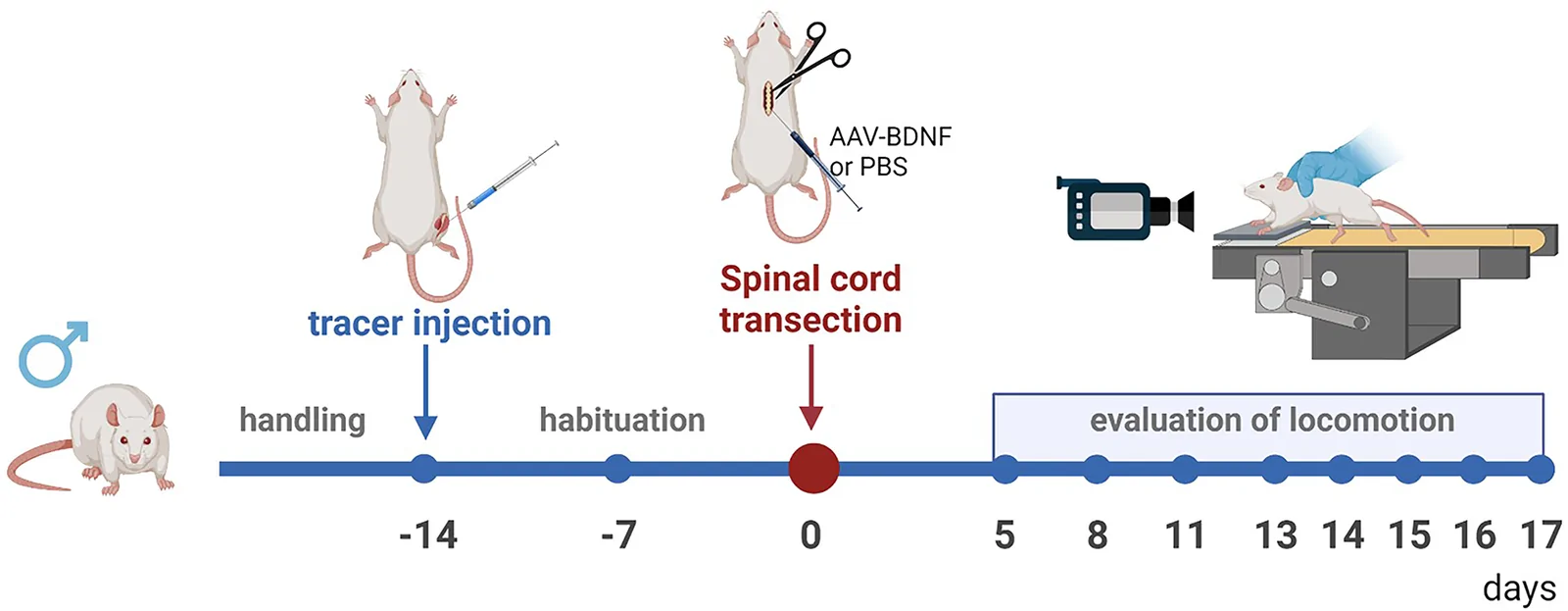
Flowchart summarizing the experimental procedures. Created with BioRender.com.

The study was performed on 47 adult male Wistar rats weighing 260–350 g upon arrival. This cohort was divided into six groups: control group 1 (*N* = 9), SCT-PBS group 1 (*N* = 7) and SCT-BDNF group 1 (*N* = 7), which were used to assess mRNA expression levels in tissue samples; control group 2 (*N* = 8), SCT-PBS group 2 (*N* = 8) and SCT-BDNF group 2 (*N* = 8), which were used to assess in situ mRNA in MNs and protein expression. Animals originated from an outbred colony (International Laboratory Code Registry: Cmdb: Wi) of Wistar rats (RRID_RGD_13508588) and were purchased from the Medical University of Białystok (Białystok, Poland). They were housed in the animal facility of the Nencki Institute of Experimental Biology in Warsaw, with free access to water and standard pellet food, under standard humidity and temperature conditions, on a 12 h light-dark cycle. Rats were housed in groups of 4–6 prior to surgery and were housed individually postoperatively. Experimental protocols involving animals, their surgery and care were approved by the First Local Ethics Committee in Warsaw ( Nos 535/2014 and 782/2015) and were conducted in compliance with the guidelines of the European Community Council Directive of 24 November 1986 (86/609/EEC), the directive of 22 September 2010 (2010/63/EU) and the Animal Protection Act of Poland (2017) concerning the use of animals for scientific purposes. An overview of the experimental procedures is presented in Fig. 1.

Neurotracers were bilaterally injected into the extensor (Gastrocnemius Lateralis, GL) and flexor (Tibialis Anterior, TA) muscles. Two weeks later, a complete spinal cord transection was performed at the thoracic Th9 level, followed by injection of either AAV-BDNF or PBS into the lumbar L1 segment. Locomotor performance of SCT rats injected with PBS (SCT-PBS) was evaluated on a running treadmill on days 5, 8, 11, and 13 post-operation (DPO). For AAV-BDNF–treated SCT rats (SCT-BDNF), locomotor performance was additionally assessed on days 14, 15, 16, and 17 DPO.

### Retrograde tracing of motoneurons

Two weeks before spinal cord transection, the animals received a subcutaneous (s.c.) injection of butorphanol tartrate (Butomidor, 1.5 mg/300 g b.w.; Richter Pharma, Wels, Austria) as an analgesic premedication, followed by anesthesia with isoflurane (1–2.5% in oxygen; Baxter, Lessines, Belgium) administered via a facemask. The skin overlying the selected muscles was shaved and disinfected with 3% hydrogen peroxide at the incision sites over the bellies of the GL, Sol and TA muscles. Cholera toxin conjugated to Alexa Fluor 555 or Alexa Fluor 488 (0.01% solution in phosphate buffered saline, Molecular Probes, US), and Fast Blue (2% aqueous solution, Dr. Illing Plastics GmbH, Germany) were injected bilaterally into the GL, Sol and TA muscles, respectively, to label MNs innervating these muscles (Fig. 1). Needle placement within the muscle belly was carefully monitored using magnifying glasses. The injection lasted around 10 min ; the needle was left in the muscle for at least 5 min after the injection was completed to avoid tracer leakage. The injection site was cleaned and the skin sutured. The analgesic Tolfedine (tolfenamic acid 4%, 4 mg/kg, s.c.; Vetoquinol, Lure Cedex, France) was given five days postoperatively, and Baytril (Enrofloxacinum 5 mg/kg; Bayer GmbH, Leverkusen, Germany) was given for five consecutive days to prevent infection. Plastic collars (Harvard Apparatus, Holliston, Massachusetts, US) were placed on each animal for two days postinjury to protect their wounds from licking, and rats were returned to individual cages with *ad libitum* access to water and food. All rats were subjected to intramuscular injections of tracers as described above. Among them, thirty animals were subjected to subsequent complete spinal cord transection together with intraspinal injections of either of PBS (*N* = 15, SCT-PBS group) or the AAV-BDNF vector (*N* = 15, SCT-BDNF group) below the lesion site. The control group (*N* = 17) consisted of non-lesioned rats.

### Spinal cord transection and AAV-BDNF or PBS intraspinal injections

Surgical procedures (Fig. 1) were performed as previously described [11]. Briefly, the animals were given a s.c. injection of butorphanol tartrate (Butomidor, Richter Pharma, Wels, Austria; 3.3 mg/kg) as a premedication and then anesthetized with isoflurane (Baxter, Lessines, Belgium, 1–2.5% in oxygen) via a facemask. The first laminectomy was performed at the thoracic Th9 vertebrae, and the cord was completely transected. The second laminectomy was performed at the Th11 vertebrae to expose the spinal cord surface at the lumbar L1-2 segments for injections. After opening the dura and pia maters AAV-BDNF or PBS solutions were injected via a fine glass capillary, inserted into the spinal cord bilaterally, about 0.7 mm lateral to the midline, at a depth of 1 mm. A surgical stereomicroscope Nikon SMZ 1000 was used to control positioning and movement of the capillary and avoid bending of the spinal cord. One microliter of either a viral particle solution (3.6 × 10^8^ AAV particles) or PBS was injected at a rate of 0.1 µL per minute (sp101i syringe pump, WPI, Sarasota, FL, US). Injections were given within 30 min following spinal cord transection.

Five minutes after injection, the capillary was removed, the cut tissues were sutured, the skin over the wound was closed with sterile stainless-steel staples, and 5 mL of 0.9% NaCl was injected. The antibiotic Sultridin (30 mg/kg, Norbrook, Ireland) and an analgesic Vetaflunix (2.5 mg/kg, s.c., VET AGRO, Poland) were administered for five postoperative days. Immediately after the surgery, the rats were placed individually in warm cages and monitored until full recovery from anesthesia. The animals were observed for their general condition, cleaned and subjected to manual bladder expression twice daily. No significant health problems were observed throughout the experiments apart from occasional bladder bleeding during the initial post-surgery days. Spontaneous micturition returned in the second week after surgery.

### Evaluation of treadmill locomotion using modified BBB scale and open field observations

The locomotion of the SCT-PBS and SCT-BDNF rats was examined on a running treadmill. The Basso-Beattie-Bresnahan (BBB) scale for evaluation of quadrupedal locomotion, modified by Antri and co-workers (mBBB) [29, 30] was used. This scale allows for evaluation of hindlimb movements on a moving treadmill when assisted by the experimenter. Five days before surgery, rats were accustomed to walking on the treadmill twice daily, as described previously [11, 26]. Locomotor assessments of spinalized rats began five days after surgery. Both forelimbs and the rostral trunk were positioned on a platform elevated 1 cm above the treadmill belt, while the hindlimbs were placed on the running treadmill. An experimenter restrained lateral and vertical movements of the trunk and secured the proper position of the trunk and forelimbs during testing. All rats were examined while walking on the treadmill at a speed of 0.05 m/s during two 2-min sessions with a 1 min break in home cages. All sessions were recorded with a digital camera (Panasonic NV- GS400) at 25 frames/s. The camera was placed perpendicularly to the longitudinal axis of the rat’s body, so that the side views of the left and right hindlimb (1 min each) were recorded during the session. To monitor the timeline of motor recovery after BDNF or PBS treatments, locomotion of the rats was examined on days 5, 8, 11, and 13 post-operation (DPO), and in the SCT-BDNF group this examination was additionally performed on DPO 14, 15, 16, and 17. The locomotion sessions were followed by a 1 min observation in the open-field. Animals were scored according to the frequency of hindlimb movements (0, no movement; 1, low-frequency movement; 2, frequent movement comparable to the forelimbs; and 3, very frequent movement exceeding the forelimbs). This timetable was chosen because in our previous study it was shown that BDNF overexpression in the lumbar segments improved locomotor performance of spinal rats on a running treadmill at an early time point (~ 2 weeks), with slower and less stable improvement observed up to 6–7 weeks [11].

### Tissue preparation and Laser Microdissection (LMD) of MNs

Rats were deeply anesthetized with a lethal dose of Morbital (pentobarbital 120 mg/kg, Biowet Puławy Ltd., Poland) and perfused transcardially with 250 mL ice-cold 0.01 M phosphate-buffered saline (PBS: 154 mM NaCl, 1.3 mM Na_2_HPO_4_, 2.5 mM NaH_2_PO_4_, pH 7.4). The vertebral column was excised and placed on ice. The lumbar (L) 3–6 segments of the spinal cord were rapidly dissected in a cold room, immediately placed in dry ice-precooled tubes and stored at -80 °C until sectioning.

The frozen L3-6 segments were surrounded by Jung tissue-freezing medium (Leica, Nussloch, Germany, cat no 14020108926) and cut into 25 μm thick, longitudinal sections on a cryostat (Leica CM1850) at -20° C. The sections were mounted onto RNase-free PET-Membrane Frame Slides (Leica, No. 11505190), with 4 sections/slide. Slides were immediately placed in a dry ice precooled box and stored at -80 °C. Prior to LMD, slides were dehydrated through a graded ethanol series: 70%, 80%, 90% and 2 × 100%, for 30 s each. After dehydration sections were cleared in two washes of 100% xylene (30 s and 180 s, respectively) and air dried for 3–5 min. MNs were isolated using the Leica Laser Microdissection system LMD7000.

For LMD, RNase-free caps of 0.2 mL tubes were placed on the tube holder for sample collection and pre-filled with 20 µL of extraction buffer (from the Arcturus™ PicoPure™ RNA Isolation Kit, KIT0204, Applied Biosystems). The air-dried slide was placed on the slide holder of the LMD 7000 system. Under the LMD system microscope (x10, 0.32 NA and x63, 0.7 NA objectives) attached to the software unit, MNs were identified, selected and then cut with a UV laser. After the collection was completed for one slide, the microfuge tube was removed from the holder. The total number of cells per group amounted to about 300 MNs and was comparable between groups (Supplementary Fig. 1). Collected MNs were lysed by incubating the sample in an extraction buffer for 30 min at 42 °C. The lysates were centrifuged for 2 min at 800 x g to collect them at the bottom of the tubes. The tubes were then snap-frozen on dry ice and stored at -80 °C. To minimize RNA degradation, each slide was processed within 60 min.

### RNA isolation from microdissected MNs and qPCR

The total RNA from the GL, Sol and TA MNs was extracted using the Arcturus PicoPure™ RNA Isolation Kit (Thermo Fisher Scientific, No. KIT0204) according to the manufacturer’s instructions. RNA extracted from Sol samples had unsatisfactory quality; therefore, that material was not included in the study. RNA was then pre-amplified and reverse-transcribed using a QuantiTect^®^. Whole Transcriptome kit (Qiagen, Nos. 207043 and 207045). The final concentration of the cDNA was determined by utilizing the Quant-iT™ PicoGreen™ dsDNA Assay Kit (Thermo Fisher Scientific, No. P7589). RT-qPCR was used to analyze gene expression levels with glyceraldehyde-3-phosphate dehydrogenase (*Gapdh*) as an internal control gene. Probes and primers are listed in Supplementary Table 1. The RT-qPCR reactions were performed in a 20 µL reaction mixture containing 7.4 µ L of PCR-grade H_2_O, 10 µL of LightCycler 480 Probes Master solution (Roche, cat no. 04887301001), 0.4 µL each of forward primer (20 µM) and reverse primer (20 µM) for the target gene, 0.4 µL probe (10 µM) for the target gene, 0.1 µL forward and reverse primer (20 µM each) for the *Gapdh* gene, 0.2 µL of probe (10 µM) of the *Gapdh* gene, 1 µL of diluted cDNA sample. A Roche LightCycler 96 was used to run the reactions with the following thermal cycling profile: preincubation at 95℃ for 10 min, 65 cycles of denaturation at 95℃ for 10 s, annealing at 60℃ for 10 s, and extension at 72℃ for 10 s. Data were analyzed using the LightCycler software. The 2^−ΔΔCt^ method was used for relative quantification of gene expression levels based on the target and reference genes’ Ct (cycle threshold) value, defined as the cycle number at which the amplification curve reaches the threshold line.

### Tissue preparation for fluorescent in situ RNA hybridization and immunofluorescence

Rats were deeply anesthetized with a lethal dose of Morbital (pentobarbital 120 mg/kg, Biowet Puławy Ltd., Poland) and perfused transcardially with 250 mL ice-cold 0.01 M PBS, followed by 4% paraformaldehyde in 0.1 M PB for 20 min. The isolated blocks of spinal cord segments were further fixed in a 4% paraformaldehyde solution in 0.1 M PB for approximately one hour at 4 °C and cryoprotected for several days in buffered sucrose solutions of increasing concentrations at 4 °C, until they were saturated. The tissue was stored in a 30% sucrose solution at 4 °C.

### RNAscope fluorescent in situ RNA hybridization combined with immunofluorescence

The RNAscope^®^ fluorescent in situ hybridization method (ACDBio, CA, USA) employed in this study is an advanced hybridization technique based on Tyramide Signal Amplification (TSA) that enables single-molecule RNA detection, visible under a microscope as a distinct punctate signal. In tissue sections, the number of visible puncta was used as a relative measure of target RNA abundance and was not interpreted as an absolute count of individual RNA transcripts. Two independent probes, called “Z-pair probes,” hybridize with the target sequence in tandem, allowing signal amplification to occur. Given the low likelihood of two independent probes binding nonspecifically in close proximity, signal amplification ensures high specificity to the target sequence. An additional advantage of this method is its effective hybridization of even partially degraded RNA, owing to the short target sequence of the Z-pair probe (40–50 nucleotides). Wang et al. [31] have confirmed the applicability of this method for sensitive RNA detection and visualization of individual RNA transcripts under optimized conditions. In the present spinal cord tissue-based analysis, puncta may vary in size and shape, and closely located signals may not always be resolved as separate RNA molecules, therefore, the observed puncta were treated as RNAscope signals reflecting relative RNA abundance. The combination of fluorescent in situ hybridization with immunofluorescence provided information on the cellular and spatial distribution of transcripts encoding key components of BDNF signaling. Probes utilized for the RNAscope method are listed in Supplementary Table 2.

Transverse sections of the spinal cord were used to analyze the localization of *Bdnf* and *Ntrk2* transcripts together with the c-Myc tag of transgenic BDNF (mouse Ab, 9B11, 1:500, Cell Signaling Technology, Danvers, MA, USA) and endogenous BDNF protein (rabbit Ab, ANT-010, 1:200, Alomone Labs, Jerusalem, Israel). Hybridization and amplification steps were performed according to the RNAscope^®^ Multiplex Fluorescent Kit v2 User Manual provided by ACD (https://acdbio.com/ technical-support/user-manuals), in HybEZ™ II Oven with a temperature-controlled humidifying chamber. The RNAScope procedure was followed by a standard immunofluorescence reaction. The s ections were washed (3 × 5 min) in PBS and incubated overnight at 4 °C with primary antibodies in PBS. The following day, spinal cord sections were washed (3 × 5 min) in PBS and incubated with the appropriate secondary antibodies for 1 h at room temperature. Next, the sections were washed (3 × 5 min) in PBS, counterstained with DAPI, mounted onto glass slides, air-dried, and cover slipped with Mowiol 4–88 medium. To check for non-specific binding of secondary antibodies to the tissue, the primary antibodies in the incubation medium were omitted. Primary and secondary antibodies used in the study are listed in Supplementary Table 3.

### Confocal microscopy and image acquisition

The Zeiss LSM780 confocal microscope was used to collect images of IF reactions. The PL APO 63x / 1.4 Oil DIC lens was used. Stacks of digital images with dimensions of 2048 × 2048 pixels, with a pixel size of 0.05 μm, sampled in the z axis every 0.21 μm were collected. Since the perikarya of α-MNs are large, t issue sections typically contain only a portion of a single MN within 25 μm thickness. Image stacks consisted of 60–64 optical sections, representing a cell body fragment of about 13 μm in the Z axis. An argon and UV laser was used to induce fluorescence. Constant exposure parameters were maintained for all four channels used for signal acquisition.

### Image analysis - quantification and spatial localization of RNAscope signals in motoneurons

For transcript level analysis in labeled MNs, fluorescence microscopy images were analyzed, and mRNA punctate signals within the cell were counted using ImageJ software. Nuclear and cytoplasmic signals were distinguished. Transcript level analysis was conducted as follows: (1) only MNs with nuclei clearly visible along the Z-axis were selected; (2) for each MN, a 10-Z-stack was cropped: five optical sections above and five below the region with the largest nuclear diameter were selected; (3) mRNA punctate signals were counted following the manufacturer’s instructions, using the same analysis criteria for all experimental groups, and disregarding variability in signal intensity .

### Study design and statistical analysis

The sample sizes used in the study were based on our previous experience with similar studies [8, 20] and were larger than those in our long-term study [11], consistent with those employed in the field. The animals were randomly assigned (1:1 allocation) to the control and treatment groups as they became available. Behavioral testing was performed and video-recorded by two investigators (B.J. and A.G.). The recordings were subsequently analyzed and quantified by B.J., A.G., and J.Cz.B., with J.Cz.B. remaining blinded to the SCT treatment groups. The STATISTICA 13.1 software (StatSoft Inc, Tulsa, OK, US) was used to analyze the data.

To test significance of the differences between SCT-PBS and SCT-BDNF groups in locomotor performance on the running treadmill at Day 13, a two-sided Fisher exact test using the presence or absence of step-like hindlimb movements as categorical endpoint was used. For assessment of the differences between groups in hindlimb movement frequency in the open field the χ² test and Fisher’s exact test were used.

Outliers were identified using Grubbs’ test and subsequently removed from the dataset (https://www.graphpad.com/quickcalcs/Grubbs1.cfm*).* Data normality and homogeneity of variance were verified using the Shapiro-Wilk test and Levene’s test, respectively. One-way ANOVA followed by Tukey’s post hoc test was used for the analysis of RNAscope data for BDNF and TrkB expression. For non-normally distributed data, the Mann-Whitney U-test was used to compare two independent groups. The Wilcoxon signed-rank test was employed for within-group comparisons between dependent samples (TA and GL MNs).

To examine relationships among gene expression profiles within the GL and TA MN population, correlation analyses were carried out using the classical Pearson test for every pairwise combination of all genes (corr.test R package), as described previously [8]. The heat-mapped correlograms were produced using the corrplot package under R with a scale from − 1 to 1. The correlograms were generated for GL and TA MNs from Control, SCT-PBS and SCT-BDNF groups to identify changes in the co-expression pattern of all gene pairs under study. The scatter plots and bar graphs were drawn using GraphPad Prism 10.0 software and CorelDraw software. P-values less than 0.05 were considered statistically significant.

## Results

### Immediate AAV-BDNF treatment after SCT evokes treadmill locomotion with body weight support

**Fig. 2:**
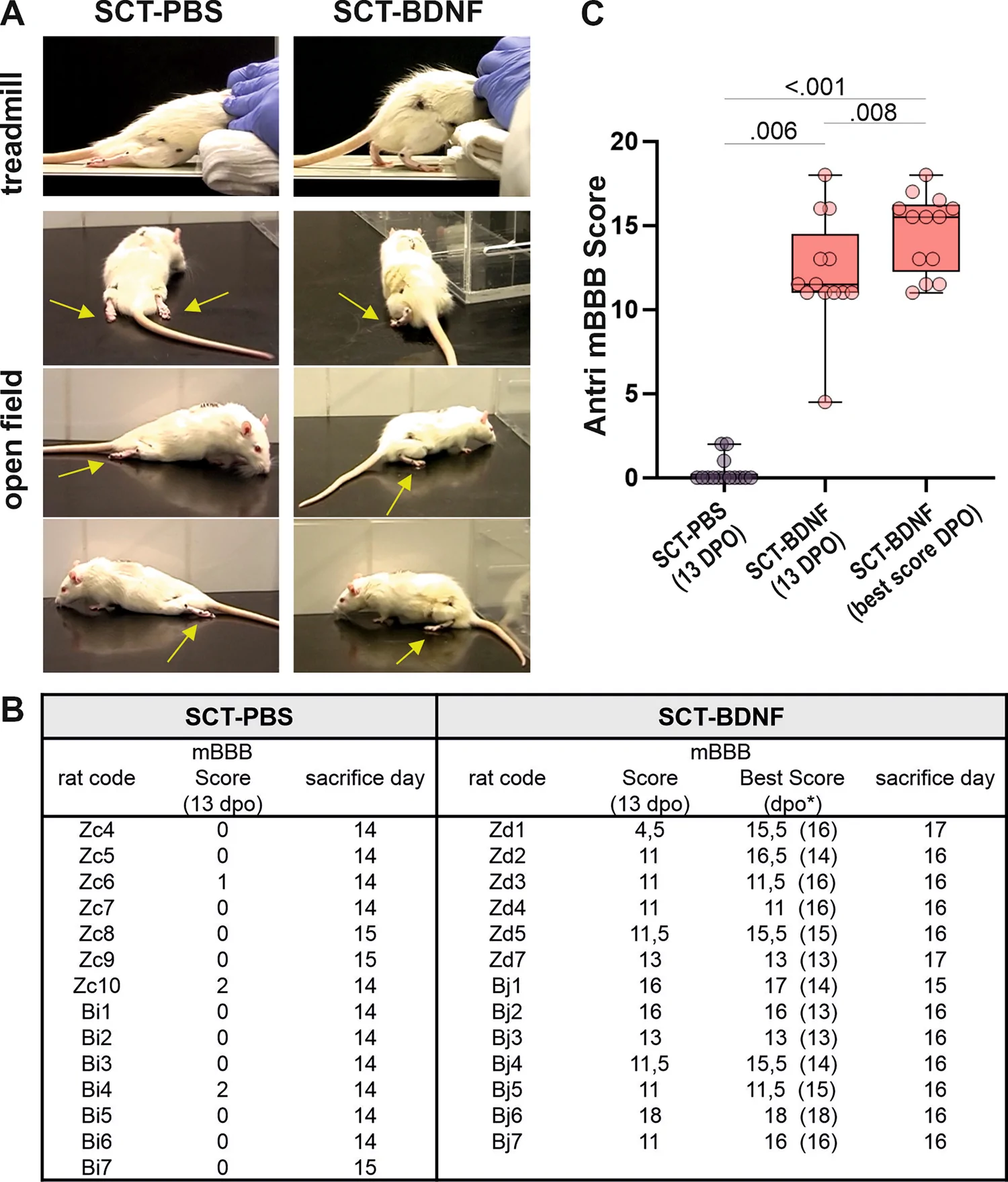
AAV-BDNF treatment administered immediately after SCT improves locomotion. **(A)** Representative locomotor performance of PBS (Bi3, 13 DPO) and AAV-BDNF (Zd5, 13 DPO) treated spinal rats on a running treadmill and in the open field. In the open field, a typical foot position during movement is shown (yellow arrow): the SCT-PBS rat drags its hindlimbs with feet in dorsal position, while the AAV-BDNF-treated rat demonstrates active hindlimb movement and plantar foot placement, enabling walking on the treadmill. **(B)** mBBB scores. **(C)** Mean mBBB scores per group. Data are presented as box-and-whisker plots, where the horizontal line represents the median, the box indicates the interquartile range and the whiskers show the minimum–maximum values. A significant difference was observed between SCT-PBS (*N* = 14) and SCT-BDNF (*N* = 13) rats on the 13th day after treatment (Mann-Whitney U test; *p* = 0.006), demonstrating that AAV-BDNF significantly enhances locomotor function in spinalized rats during treadmill walking. In most SCT-BDNF rats, motor performance continued to improve over the following days (*14–18 DPO) reaching significantly higher scores by day 16 compared to day 13 (Wilcoxon test, *p* = 0.008)

We first video-recorded and evaluated the motor abilities of SCT rats on a running treadmill (Fig. 2A, upper row), followed by open field testing (Fig. 2A, middle and bottom rows). No pressure stimulation of the tail was applied during testing in both conditions. Treadmill locomotion was evaluated by three experienced observers who independently scored each rat’s behavior following the standardized protocol. Discrepancies in scores were reviewed and resolved by consensus.

The rats walking on the treadmill showed marked group differences. All SCT-PBS rats, tested on the 13th DPO, walked on the dorsal surface of their paws and were unable to support their body weight (Fig. 2A, upper left image, Supplementary Table 4). Several spontaneous hindlimb movements occurred, but no alternative movements were detected. In contrast, SCT-BDNF rats at the same time point displayed step-like hindlimb movements with right-left alternation, plantar foot placement, and body weight support (Fig. 2A- upper right image). Quantitative analysis of bipedal treadmill locomotion was performed using mBBB scale [29, 30], which consist of four levels and 22 scores: level 1 (scores 0–1), level 2 (scores 2–9), level 3 (score 10), and level 4 (scores 11–22), reflecting increasing motor capabilities. A score of 0 at level 1, indicates no movement, whereas score 22 at level 4 reflects normal locomotion. On day 13 after SCT, twelve of fourteen SCT-PBS rats scored at level 1, while the remaining two reached level 2. In contrast, twelve out of thirteen SCT-BDNF rats reached level 4. One rat was classified at level 2 with a score of 4.5, still exceeding the performance of any SCT-PBS rat (Fig. 2B). Mean treadmill locomotion scores on day 13 were 0.36 ± 0.74 in SCT-PBS rats versus 12.19 ± 3.31 in SCT-BDNF rats (Mann-Whitney U test, *p* = 0.006) (Fig. 2C), demonstrating that BDNF overexpression significantly improved locomotion of spinal rats on the running treadmill, with a progressive effect. A two-sided Fisher’s exact test using the presence or absence of step-like hindlimb movements at Day 13 as a categorical endpoint also showed a highly significant difference between groups (*P* < 0.0001). Variation in SCT-BDNF scores on day 13 suggested that some rats (e.g., *Bj1*,* Bj2*,* Bj6*, Fig. 2B) had reached their highest level of motor performance, while others (e.g., *Zd7*,* Bj3*,* Bj7*) had not. To assess the possibility of further improvement, the observation of SCT-BDNF rats was prolonged by 4 additional days. Indeed, by day 16, most had improved, reaching level 4 with a mean score of 14.62 +/- 2.32 (significantly higher than day 13, *p* = 0.008, Wilcoxon test) (Fig. 2C). These results demonstrate a beneficial effect of AAV-BDNF treatment on motor recovery during the early post-SCT period.

In the open field, SCT-PBS rats moved using their forelimbs while dragging the body, either symmetrically or with lateral bending on the surface of the ground. Their hindlimbs were dragged on the dorsal aspect of the feet and displayed only sporadic movements (Fig. 2A, lower left panel). The AAV-BDNF treatment enabled step-like hindlimb movements, with plantar foot placement, occasional right-left alternation, and sporadic body weight support (Fig. 2A, lower right panel).

Open field observations provided complementary evidence of improved hindlimb motor activity following BDNF treatment. Animals were observed for 1 min and scored according to the frequency of hindlimb movements (0, no movement; 1, low-frequency movement; 2, movements with a frequency comparable to that of the forelimbs and 3, movements with a frequency exceeding that of the forelimbs). For categorical analysis, scores 0–1 were classified as low hindlimb activity, whereas scores 2–3 were classified as frequent hindlimb activity. In the SCT-PBS group, 5 of 7 animals (71%) scored 1, while 2 animals scored 0. In contrast, 10 of 13 SCT-BDNF animals (76.9%) reached scores 2–3, while 3 animals (23.1%) scored 1. The distribution of low versus frequent hindlimb activity differed significantly between treatment groups (χ²(1) = 7.21, *p* = 0.0072; Fisher’s exact test, *p* = 0.0166). Asymmetric hindlimb positioning was observed in 7/12 SCT-BDNF animals, whereas all SCT-PBS animals showed symmetric hindlimb positioning, reflecting the absence of detectable hindlimb activity. Plantar foot placement, alternative hindlimb stepping-like movements, and episodes of body weight support were observed only in the SCT-BDNF group indicating increased hindlimb motor activity following BDNF treatment (Supplementary Table 4) also during spontaneous walking in the open field.

### Immediate AAV-BDNF treatment after SCT increased spinal BDNF expression and restored TrkB transcript levels in hindlimb MNs two weeks after surgery

**Fig. 3:**
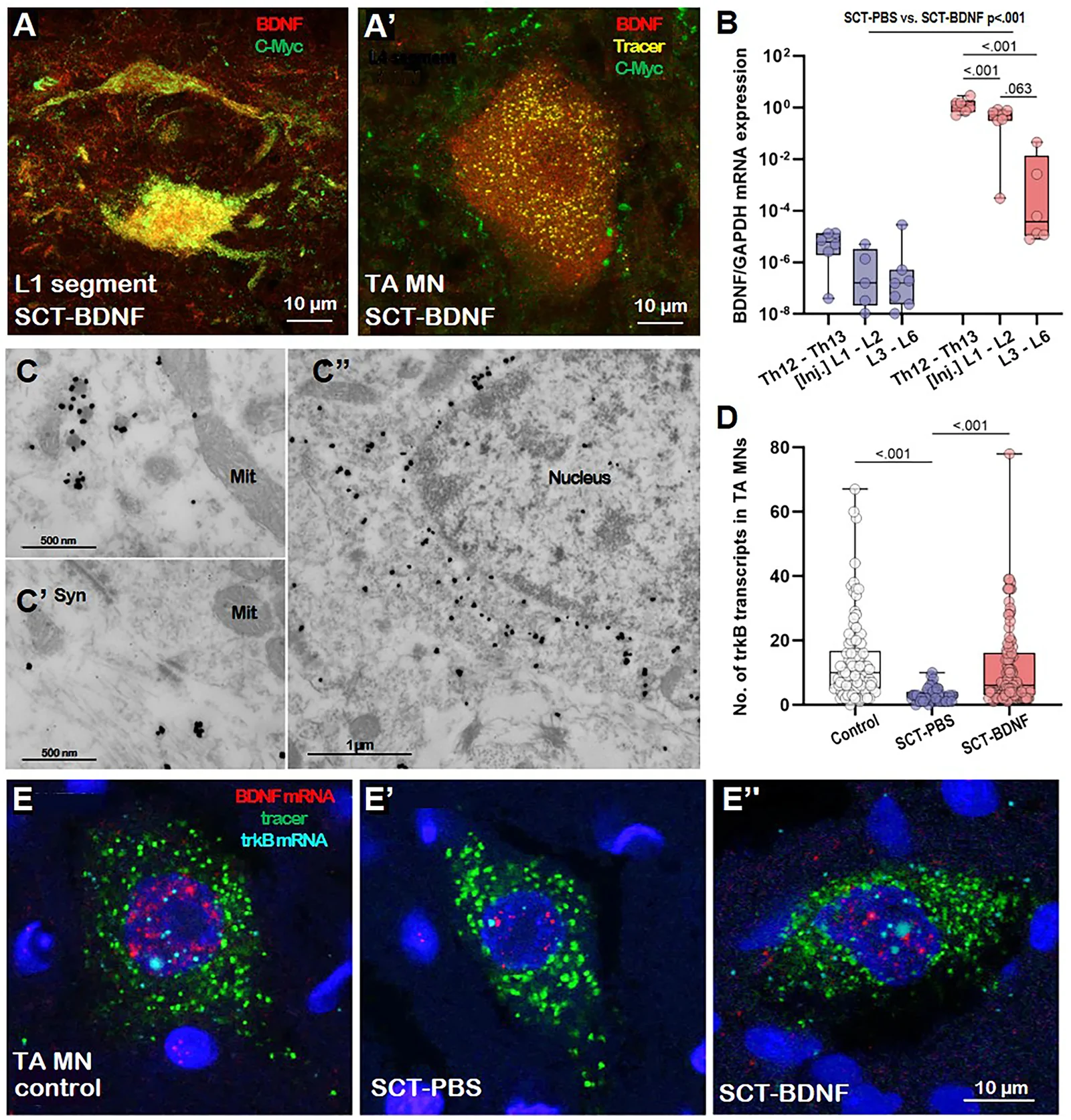
A-C. Evaluation of AAV-mediated BDNF expression in the rat spinal cord using IF, TEM and RT-PCR. (**A**) Representative confocal microscope images. In the L2 segment, BDNF protein is visible in the cytoplasm of neurons and their processes. Recombinant c-Myc–tagged BDNF shows a similar distribution. In the L4 segment, c-Myc immunoreactivity is observed in fibers surrounding tibialis anterior motoneuron (TA MN), identified with neurotracer and expressing BDNF. (**B**) BDNF transcript levels in SCT-PBS and SCT-BDNF groups in spinal cord segments Th12–L6. Data are presented as box-and-whisker plots, where the horizontal line represents the median, the box indicates the interquartile range and the whiskers show the minimum–maximum values. Data were analyzed using two-way ANOVA followed by Tukey’s multiple comparisons test. (**C**) Representative transmission electron microscope (TEM) images showing localization of recombinant BDNF tagged with c-Myc, detected via immunogold labeling in the L1 segment. The signal is present in electron-dense secretory vesicles (**C**), adjacent to synapses (**C’**), and in clusters surrounding the cell nucleus **(C’’**). **(D**-**E) **Evaluation of BDNF and TrkB RNAscope signals, reflecting relative mRNA abundance, in motoneurons innervating the tibialis anterior (TA) muscle.** (D**) Quantification of TrkB RNAscope puncta per TA motoneuron in Control, SCT-PBS and SCT-BDNF rats. Each dot represents a single motoneuron. Outliers were identified using the Grubbs test (α = 0.01) and excluded from statistical analysis (one outlier (value = 78) in the SCT-BDNF group). Data are presented as box-and-whisker plots, where the horizontal line represents the median, the box indicates the interquartile range and the whiskers show the minimum–maximum values. Differences among groups were analyzed using the Kruskal–Wallis test followed by Dunn’s multiple-comparisons test. (**E**) BDNF and TrkB mRNA signals are predominantly localized to the nucleus of TA motoneurons, with additional puncta observed in the cytoplasm and adjacent oligodendrocytes. TrkB mRNA signals appear as paired nuclear foci, potentially representing expression from both alleles, and as single cytoplasmic puncta. Images are single optical sections acquired using a Leica SP8 confocal microscope with a 63× oil-immersion objective

Behavioral testing showed improved locomotor performance in the SCT-BDNF group. To assess transgene- mediated BDNF expression, we quantified BDNF transcript and protein at the segmental, cellular and subcellular levels (Fig. 3A-C).

AAV-BDNF administration resulted in significantly higher levels of BDNF transcripts (endogenous and recombinant) in the thoracic (Th12–13) and lumbar (L1–L2 and L3–L6) spinal cord segments compared with PBS-treated animals (Fig. 3B). In SCT-PBS rats, mean BDNF mRNA levels were 7.00 × 10⁻⁶ ± 5.58 × 10⁻⁶ in Th12–Th13, 1.34 × 10⁻⁶ ± 2.18 × 10⁻⁶ at the injection site (L1), and 4.23 × 10⁻⁶ ± 1.08 × 10⁻⁵ in L3–L6. In SCT-BDNF rats, the corresponding values were 1.35 ± 0.857, 0.490 ± 0.295, and 8.03 × 10⁻³ ± 1.84 × 10⁻², respectively (Mean values ± SD). The overall treatment effect was significant (F 1,31 = 25.7, *p* < 0.001, two-way ANOVA followed by Tukey’s multiple comparisons test) (Fig. 3B). Recombinant BDNF was abundant in neuronal bodies in L2 and in fibers abutting MNs in L2-L4 (Fig. 3A, A**’**), suggesting local availability of AAV-derived BDNF to lumbar MNs [20].

We next used RNAscope to determine whether BDNF overexpression affected the abundance and cellular localization of BDNF and TrkB mRNA in tracer-identified lumbar MNs. Representative data from TA MNs are shown on Fig. 3D**-**E. In SCT-PBS rats, BDNF mRNA levels were largely preserved compared with controls (data not shown). In contrast, TrkB mRNA signal was reduced across Sol and TA MNs following SCT (data for TA MNs shown in Fig. 3D). The mean number of TrkB RNAscope puncta per TA MN was 13.58 ± 9.93 in Control rats, 3.33 ± 2.40 in SCT-PBS rats, and 8.63 ± 10.20 in SCT-BDNF rats. Thus, SCT reduced the TrkB transcript signal fourfold (by approximately 75% ) compared with Control (*p* < 0.01), whereas AAV-BDNF treatment increased TrkB puncta by approximately 159% compared with SCT-PBS (*p* = 0.028), partially restoring the signal toward Control levels (*p* > 0.001, nonparametric Kruskal–Wallis test followed by Dunn’s multiple-comparisons test). These findings indicate that immediate AAV-BDNF treatment counteracted the SCT-associated reduction in TrkB mRNA signal in lumbar MNs two weeks following injury.

### BDNF overexpression induces alterations in GABAA (*Gabra1A*) and 5-HT2A (*Htr2a*) receptor expression in upper and lower lumbar segments following spinal cord transection

Our previous work demonstrated that AAV-mediated BDNF delivery alters the expression of presynaptic markers involved in glutamatergic (VGLUT2) and GABAergic (GABA, GAD65, GAD67) neurotransmission in lumbar spinal interneurons and reduces KCC2 expression in spinal networks at 6 weeks post-injury [11]. These findings prompted us to investigate whether postsynaptic adaptations also occur. At 2 weeks after spinalization we showed that tracer-identified MNs innervating ankle flexor and extensor muscles exhibit a marked downregulation of transcripts encoding glutamatergic receptor subunits, as well as GABAA and glycine receptor subunits and the K-Cl- cotransporter KCC2, all essential for inhibitory signaling in mature neurons, whereas 5-HT1 and 5-HT2 receptor subunits are upregulated [8]. Here, we examine whether early BDNF overexpression during the critical period for locomotor recovery enhances or prevents these changes in the expression of postsynaptic receptors and ion transporters. Given their widespread expression in the spinal cord, we first assessed the effects of AAV-BDNF treatment on transcript levels in L1-2 and L3-6 lumbar segments.

**Fig. 4:**
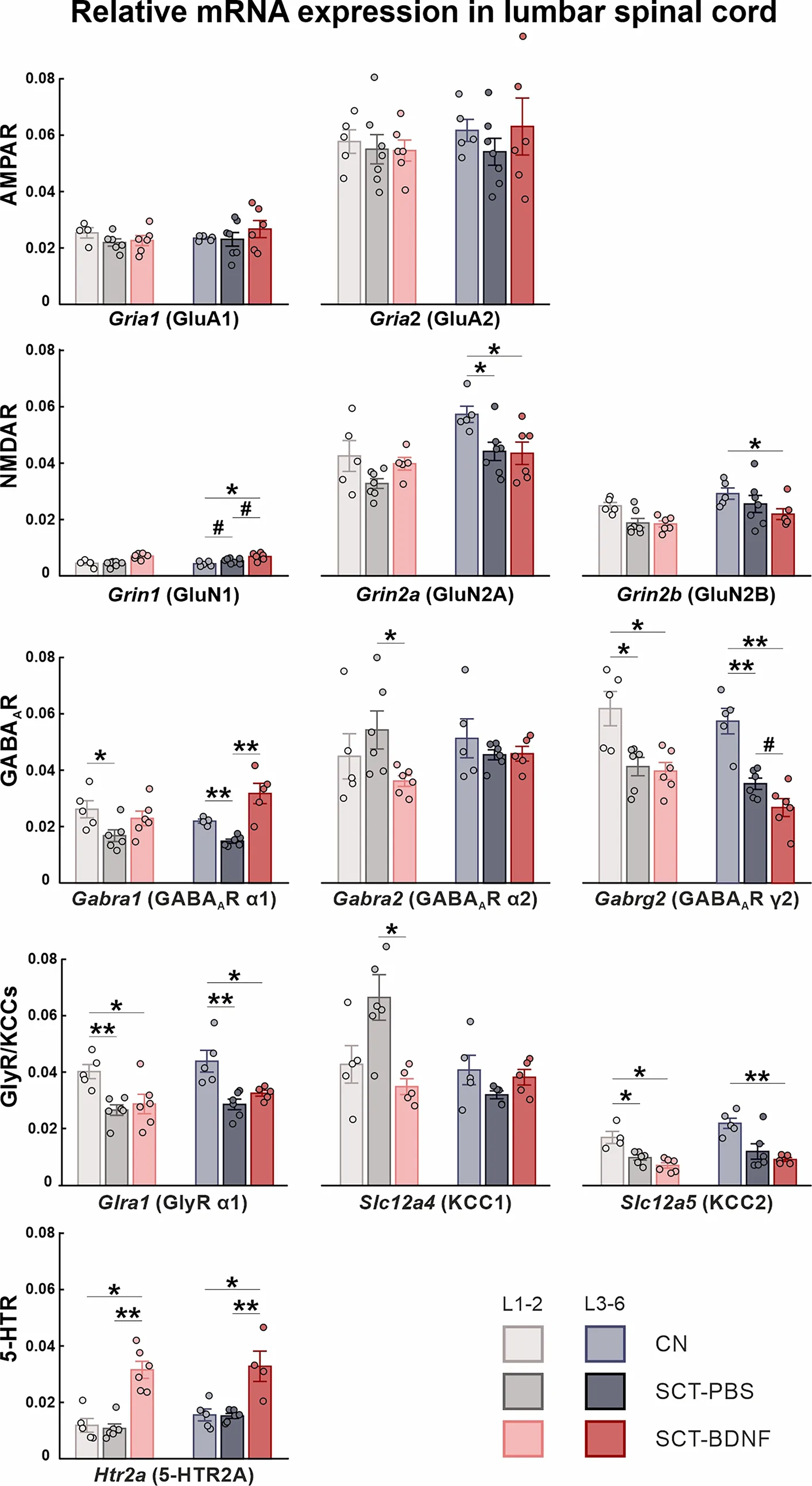
Effect of BDNF overexpression on the expression of AMPA, NMDA, GABAA, Gly, and 5-HT2A receptor subunits, and neuronal potassium-chloride KCC1 and KCC2 cotransporters expression in L1–L2 and L3–L6 spinal cord segments at 2 weeks post-injury. Data are expressed as mean ± SEM. * *p* < 0.05, ** *p* < 0.01, #*p* < 0.08; Mann–Whitney U test

In whole-segment analysis, in uninjured animals, transcript levels of the AMPA receptor *Gria2* (GluA2), NMDA receptor *Grin2a* (GluN2A), GABA receptor *Gabra2* (GABAA α2), *Gabrg2* (GABAA γ2), glycine receptor *Glra1* (GlyRα1) subunits, and *Slc12a4* (KCC2) were highest and comparable across L1–2 and L3–6 segments (Fig. 4). The similar expression levels of *Gria2*,* Grin2a*,* Gabra2*,* Gabrg2* and *Glra1* suggest a consistent distribution of glutamatergic, GABAergic, and glycinergic receptors throughout the L1–6 network.

SCT markedly reduced transcript levels of the NMDA receptor subunit *Grin2a*, but not *Grin1* and *Grin2b*, as well as those of GABAA receptor subunits *Gabra1* and *Gabrg2*, and the glycine receptor subunit *Glra1* in L3–6 segments (Fig. 4). These results confirm the post-injury decline observed in our previous study and indicate a selective downregulation of NMDAR and key inhibitory signaling components during the subacute phase of SCT. This transcriptional pattern is consistent with altered excitatory and inhibitory signaling capacity, although the net functional effect cannot be inferred from mRNA levels alone.

AAV-BDNF treatment induced specific alterations in GABAA and 5-HT2A receptor subunit expression without affecting other transcripts. The most prominent effect was a > 3-fold upregulation of *Htr2a* (5-HT2A receptor) transcripts relative to both uninjured and SCT-PBS animals, observed in both L1–2 and L3–6 segments. In the lower lumbar segments, treatment significantly upregulated *Gabra1* expression but reduced *Gabrg2* transcripts to approximately 50% of control levels. Additionally, a decrease in *Gabra2* expression was detected in the upper lumbar segments (Fig. 4).

Building on the findings that AAV-BDNF treatment alters receptor expression in a selective manner in the lumbar spinal cord, we investigated the extent to which BDNF modulates receptor expression in GL and TA MNs, functionally different motoneuron groups which control ankle muscles critical for locomotion.

### BDNF markedly changes the expression of GlyR and 5HT2A receptor subunits in gastrocnemius lateralis (GL) and tibialis anterior (TA) MNs following SCT

#### Expression of glutamatergic AMPA and NMDA receptor subunits

**Fig. 5:**
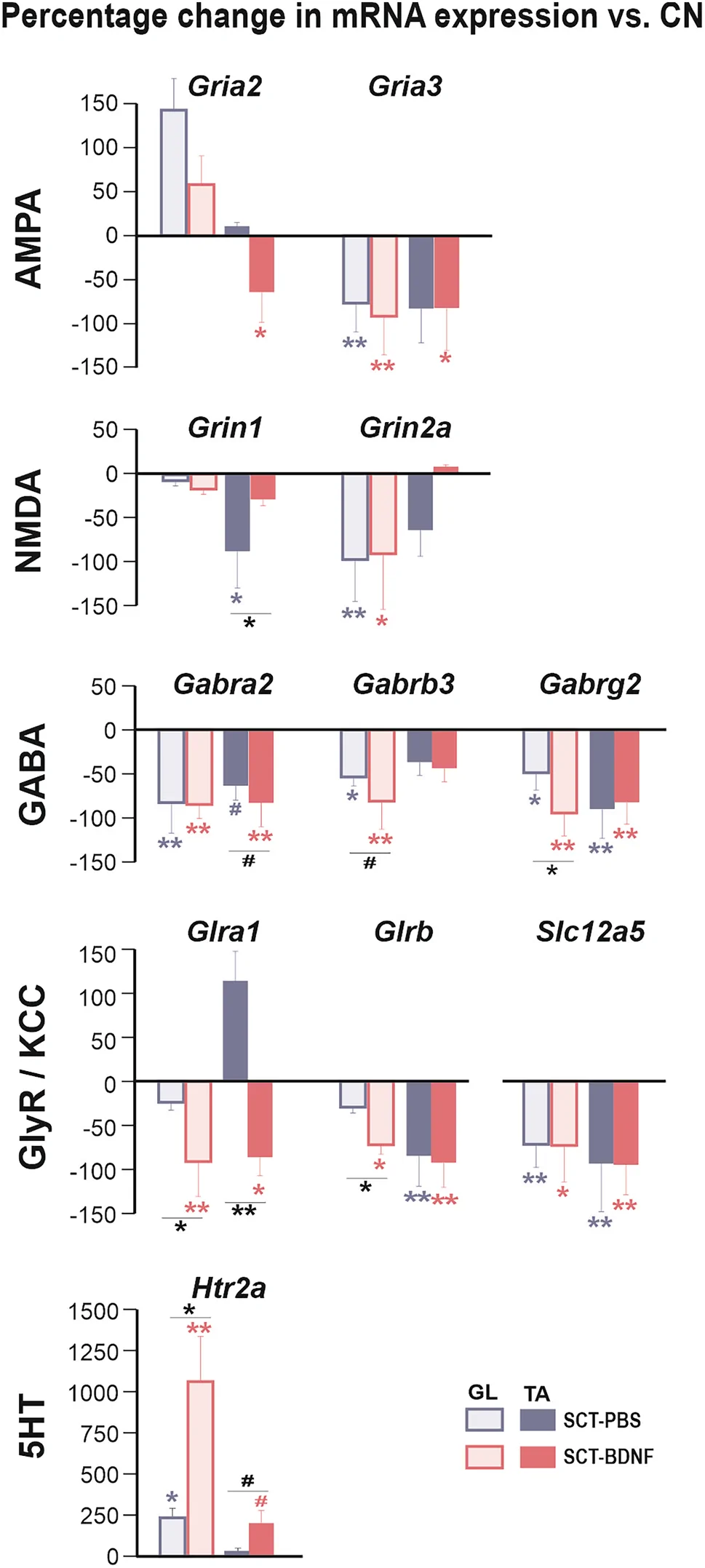
Effect of AAV-BDNF spinal injection on mRNA expression of AMPAR and NMDAR glutamatergic receptors, GABAA and Gly inhibitory receptors, theK^+^-Cl^−^ cotransporter KCC2, and the 5-HT2A receptor in gastrocnemius lateralis (GL) and tibialis anterior (TA) motoneurons 2 weeks after spinal cord transection. Data were obtained from Control (*n* = 9), SCT-PBS (*n* = 7), and SCT-BDNF (*n* = 6) rats. Values are mean ± SEM. RT-qPCR data were normalized to the GAPDH housekeeping gene and expressed as percentage change relative to the control (CN) group, calculated as (experimental group/CN − 1) × 100. Positive and negative values indicate higher and lower mRNA expression than in the CN group, respectively. The colored asterisks indicate significant differences compared with the Control (CN) group, whereas black asterisks indicate significant differences between the GL and TA motoneurons groups. * *p* < 0.05; ** *p* < 0.01; #*p* < 0.08; Mann-Whitney U-test

In the SCT-PBS group, AMPA *Gria2* mRNA tended to increase in GL MNs but remained unchanged in TA MNs. In contrast, *Gria3* was significantly downregulated in both GL and TA MNs (GL: CN vs. SCT-PBS, *p* = 0.003; TA: CN vs. SCT-PBS, *p* = 0.003; Mann-Whitney U test; Fig. 5). BDNF overexpression tended to decrease *Gria2* mRNA in both MN populations, reaching significance only in TA MNs, while *Gria3* expression remained unaffected (Fig. 5). Regarding NMDA receptor subunits, *Grin1* levels were stable in GL MNs but significantly reduced in TA MNs post-SCT (*p* = 0.03); notably, BDNF treatment reversed this reduction (*p* = 0.02). Finally, SCT significantly decreased *Grin2a* mRNA in GL MNs (*p* = 0.006) with a similar trend in TA MNs (*p* = 0.10), both of which were partially restored by BDNF overexpression (Fig. 5)

#### Expression of inhibitory receptor subunits and KCC2

In the SCT-PBS group, transcript levels of GABAA receptor subunits (*Gabra2*, *Gabrb3*, *Gabrg2*), the glycine receptor subunit *Glrb*, and the KCC2 cotransporter *Slc12a5* were significantly decreased in both GL and TA MNs (Fig. 5)

AAV-mediated BDNF delivery further reduced expression of several inhibitory components. *Gabra2* was further downregulated in TA MNs, while *Gabrb3* and *Gabrg2* were suppressed in GL MNs. A particularly strong reduction was observed at the *Glra1* expression in both GL and TA MNs and in *Glrb* levels in GL MNs. These results suggest that BDNF modulates inhibitory tone in motoneurons in a MN type- and receptor-specific manner, with strong effect on GlyR subunits following spinal injury. Whether these changes alter inhibitory synaptic transmission requires electrophysiological and protein-level studies.

#### Serotoninergic receptor subunit expression is preferentially upregulated in GL MNs by BDNF after SCT

Spinal cord transection increased Htr2a mRNA in GL MNs (*p* = 0.02) but did not affect TA MNs (Fig. 5). AAV-BDNF further elevated Htr2a expression in GL MNs (~ 4-fold, *p* = 0.02) and induced a twofold increase in TA MNs (*p* = 0.07). We also examined *Htr2b* and *Htr2c* transcripts. However, both transcripts showed low and inconsistent amplification, with no detectable signal in many samples. Because these data were not sufficiently reliable for quantitative group comparisons, we did not include them in the statistical analysis.

Conversely, noradrenergic receptor Adra1a subunit transcripts were reduced in TA MNs following SCT (*p* = 0.01) with no change in GL MNs. BDNF overexpression caused small, nonsignificant decreases in GL and increases in TA MNs (not shown).

### **Nav 1.1 sodium channel (*Scn1a*) subunit expression is downregulated in GL and TA MNs by BDNF after SCT**

**Fig. 6:**
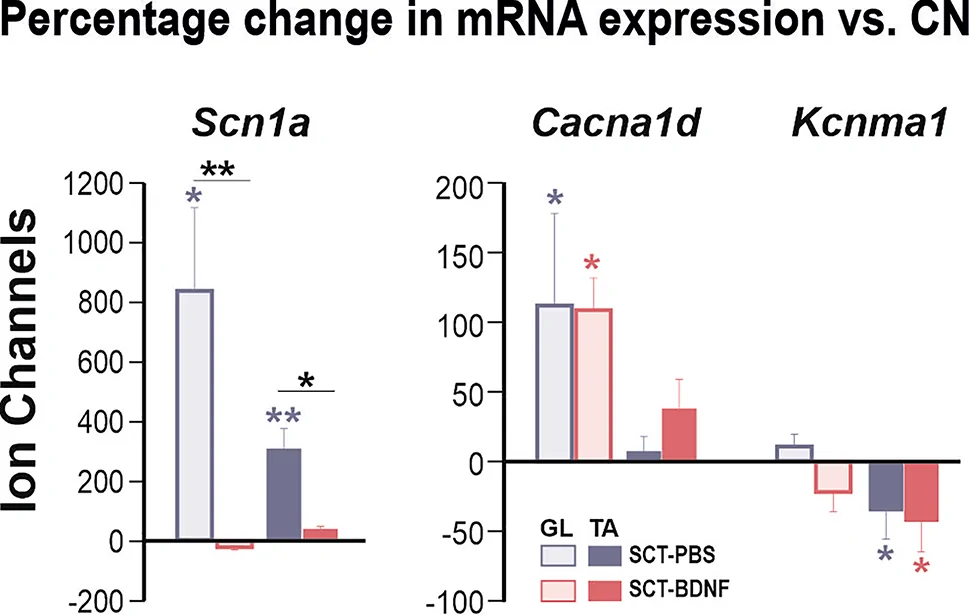
Effect of AAV-BDNF spinal injection on mRNA expression of the Nav 1.1 sodium channel(*Scn1a*), CaV1.3 calcium channel alpha subunit Cacna1d, and the Kcnma1 alpha subunit of the BK (Big K+) calcium-activated channel, in gastrocnemius lateralis (GL) and tibialis anterior (TA) motoneurons 2 weeks after spinal cord transection. Data were obtained from Control (*n* = 9), SCT-PBS (*n* = 7), and SCT-BDNF (*n* = 6) rats. Values are mean ± SEM. RT-qPCR data were normalized to the GAPDH housekeeping gene and expressed as percentage change relative to the control (CN) group, calculated as [(experimental group/CN) ^− 1^] × 100. Positive and negative values indicate higher and lower mRNA expression in spinal groups than in the CN group. The colored asterisks indicate significant differences compared with the Control (CN) group, whereas black asterisks indicate significant differences between the GL and TA motoneurons groups. * *p* < 0.05; ** *p* < 0.01; Mann-Whitney U-test

We expanded our investigation to include selected ion channels that are crucial for regulating neuronal excitability. SCT significantly elevated *Scn1a* (Nav 1.1) sodium channel mRNA levels in both GL (~ 9-fold; *p* = 0.004); and TA (~ 3-fold; *p* = 0.001) motoneurons, an effect reversed by BDNF treatment (GL: *p* = 0.01; TA: *p* = 0.02) (Fig. 6). Other ion channels showed MN-type specific changes after SCT: *Cacna1d*, (CaV1.3) increased in GL motoneurons, while *Kcnma1*, encoding the BK (Big K+) channel, was downregulated in TA motoneurons (*p* = 0.05). However, AAV-BDNF treatment had no effect on *Cacna1d* and *Kcnma1* transcript levels (Fig. 6).

SCT and BDNF overexpression were associated with altered pairwise correlations among the measured transcripts relative to Control animals (Supplementary Fig. 2). In GL MNs of Control rats, significant, positive correlations of several genes (*p* < 0.05; marked by asterisks) were not preserved in the SCT-PBS group (*Grin1* subunit of *NMDAR vs. Gria2* subunit of AMPAR, and *Glrb s*ubunit of GlyR, *Grin 2a* subunit of NMDAR *vs. Cacna 1b* calcium channel, *Gabrb3* subunit of GABAAR *vs. Scn1a* encoding the Nav 1.1 sodium channel. SCT also led to a shift toward stronger positive correlations, exemplified by *HTR2aR* and *Adra 1aR* and by *Kcnma1*, forming discrete clusters (Supplementary Fig. 2**-**GL) contrasting with more dispersed correlations in Control rats.

In the SCT-BDNF group, a treatment led to a shift toward some negative correlations. In TA MNs, gene correlations were weaker in Control rats but became more structured following SCT-PBS and SCT-BDNF (Supplementary Fig. 2**-**TA). The analysis confirmed that in Control rats, the correlated patterns of gene expression differ between GL MNs and TA MNs, as shown in our recent study for a larger set of genes [8].

## Discussion

A key novelty of our study lies in the integrated analysis of the effects of spinal cord enrichment with transgene-derived BDNF on both the neural circuits involved in locomotor rhythm generation and the motoneurons (MN), that provide the final common pathway for motor output. This integrated approach revealed coordinated molecular changes at the level of trophic signaling, neurotransmitter receptors, and ion channels and identified several potential targets through which BDNF may modify spinal motor function after spinal cord transection (SCT). Importantly, these changes were not uniformly directed toward normalization of the post-injury phenotype, but instead reflected a complex reorganization of molecular pathways controlling MN excitability.

Enhanced BDNF signaling in the spinal milieu restored TrkB expression in lumbar MNs. In parallel, BDNF induced a reorganization of gene co-expression networks linked to excitability control, including increased expression of 5-HT2A receptor transcripts and decreased expression of selected glycine and, to a lesser extent, GABAA receptor subunit transcripts. BDNF also reversed the SCT-associated increase in Nav1.1 expression. Although electrophysiological properties were not directly assessed, the coordinated regulation of these molecules is consistent with a BDNF-induced reorganization of the molecular determinants of MN excitability. This state may contribute to early altered excitability of MN during the early post-injury period and may help explain the partial restoration of locomotor function observed after BDNF treatment.

### BDNF overexpression restores locomotor function and preserves TrkB transcripts in lumbar motoneurons

AAV-mediated BDNF delivery restored TrkB receptor mRNA abundance in lumbar MNs. This intervention significantly improved treadmill locomotion, and also improved spontaneous locomotor activity in the open field, an effect not previously demonstrated under these conditions. Together, these findings indicate that increasing BDNF availability in the spinal cord can promote functional recovery after SCT and is associated with preservation of TrkB expression in lumbar MNs.

The widespread distribution of recombinant BDNF across thoracic and lumbar spinal segments in our experimental approach provides a potential explanation for the observed functional effects. BDNF was detected in neuronal soma, processes, secretory vesicles, perisynaptic regions, and perinuclear compartments. In contrast, intramuscular AAV-TrkB delivery targeting defined MN pools produced only modest locomotor improvement [32], likely because receptor overexpression was restricted to a limited subset of MNs. Together, these observations highlight that functional recovery depends not only on MN-autonomous TrkB signaling but also on BDNF actions within the broader spinal network [11].

Several lines of evidence support such a network-level action of BDNF. Muscle-derived BDNF can regulate MN excitability, as first demonstrated by Gonzales and Collins [19], and more recently supported by Grzelak et al. [33]. In a feline model of complete spinal cord injury, intrathecal delivery of BDNF to the lumbar spinal cord increased the excitability and synchrony of lumbar interneuronal populations, as reflected by elevated firing rates and enhanced spike-train coordination [34]. This shift in network dynamics was associated with improved treadmill stepping, suggesting that BDNF promotes a state of heightened spinal excitability that facilitates the integration of sensory inputs into locomotor output. Complementary evidence from intranasal delivery of BDNF-loaded small extracellular vesicles (sEV) further supports a broad neuroprotective and pro-plastic role of BDNF after acute spinal cord injury. BDNF-sEV treatment activated BDNF/TrkB signaling in the injured spinal cord, reduced glial reactivity and inflammatory signaling, enhanced neuronal survival and axonal remodeling, and delayed motoneuron atrophy below the lesion. These changes were accompanied by improved locomotor and electrophysiological outcomes in both rodent and primate models [35].

Our finding that AAV-BDNF reversed the SCT-induced reduction in TrkB transcript signal further suggests that BDNF availability may influence the maintenance of its cognate receptor after injury. Because RNAscope puncta provide a relative measure of target RNA abundance within MNs rather than an absolute count of individual RNA molecules, these data should be interpreted as differences in the relative abundance and spatial distribution of BDNF and TrkB RNA signals between experimental groups. The finding is compatible with the possibility that the selected AAV-BDNF dose produced sufficiently sustained BDNF expression to preserve TrkB transcript levels during the early post-injury period. However, the present study did not directly assess TrkB protein abundance, receptor phosphorylation, or BDNF concentration over time. Such measurements will be necessary to determine whether the treatment maintains physiological BDNF/TrkB signaling rather than simply increasing BDNF availability.

Recent evidence indicates that TrkB expression in MNs is influenced by motor unit size, with larger phrenic MNs expressing significantly higher levels of TrkB mRNA than smaller MNs [36]. Our study did not reveal such a difference between the TA and GL MN population. Although TA MNs predominantly innervate fast-twitch, larger motor units, and GL MNs comprise a mixture of motor unit types, both pools span a broad range of MN sizes, which may mask size-dependent differences at the population level. Overall, higher TrkB expression in larger motor units, which generate greater force and support fast, expulsive motor behaviors, may contribute to their functional specialization and increased resilience to injury or disease.

### Duration and dose of BDNF signaling as determinants of functional recovery

An important consideration is whether the beneficial effects of BDNF observed during the early post-injury period can be maintained with prolonged BDNF expression. The present study assessed recovery only during the first 17 days after injury. In our previous study using the same SCT model and AAV-BDNF treatment, the early improvement in stepping was not consistently maintained or further enhanced at 5–7 weeks after injury [11]. Some animals continued to improve, whereas others showed poorer locomotor performance and clonic movements at later time points, despite strongly elevated BDNF levels in the lumbar spinal cord at this stage [11]. These findings suggest that prolonged BDNF overexpression does not necessarily produce continuous functional improvement and may, under some conditions, contribute to maladaptive changes in spinal excitability.

From a therapeutic perspective, the dose, spatial distribution, and duration of BDNF exposure therefore require careful optimization. In vitro studies show that high, saturating BDNF concentrations (~ 1 nM) can induce TrkB downregulation, whereas prolonged or gradual exposure can maintain receptor availability and signaling [37, 38]. A limitation of the current study was the lack of an inducible promoter system to control AAV-BDNF expression. Future studies should determine the temporal profile and dose range of BDNF expression that best support recovery while minimizing potential adverse effects associated with prolonged or excessive TrkB activation.

### BDNF selectively modulates AMPA and NMDA receptors in lumbar motoneurons, not in the broader spinal network

BDNF overexpression decreased AMPAR subunit transcripts and increased NMDAR subunit transcripts in lumbar MNs, suggesting a shift in receptor composition compared with SCT alone. In contrast to these cell-specific effects in isolated MNs, whole-segment analysis (L1-L2 and L3-L6) did not reveal significant BDNF-induced changes in AMPAR or NMDAR subunit transcripts expression. This difference likely reflects the cellular heterogeneity of the whole spinal tissue. In whole-segment homogenates, MNs represent only a small fraction of the total cell population, which is dominated by interneurons and glial cells. Therefore, robust or cell-specific receptor adaptations occurring specifically in motor pools may be diluted or masked at the whole-tissue level. This discrepancy highlights the critical advantage of using laser microdissection (LMD) to isolate defined MN populations rather than relying solely on whole-segment tissue.

To our knowledge there are no other studies demonstrating effects of BDNF on the expression of excitatory and inhibitory neurotransmitter receptors in lumbar MNs after spinal injury. The only evidence of increased diaphragm MNs expression of glutamatergic NMDA receptors (NR1 subunit under study) in response to AAV-TrkB comes from studies that used long-term pretreatment, an approach that is not applicable in therapy. BDNF–TrkB signaling enhances phrenic MN excitability via NMDAR-dependent pathways, and intrathecal BDNF delivery improves diaphragmatic activity after cervical SCI, an effect abolished by TrkB knockdown or kinase inhibition [10, 12, 39, 40]. Intrapleural AAV-TrkB administration similarly restores ipsilateral diaphragmatic activity and increases NMDAR mRNA expression in phrenic MNs [22].

Whether BDNF–TrkB signaling similarly regulates glutamatergic transmission in functionally impaired lumbar MNs after SCI is unknown. We previously proposed that the SCT-induced shift from NMDAR to AMPAR expression may help maintain or enhance MN excitability [8]. The present findings suggest that AAV-BDNF–driven upregulation of BDNF–TrkB signaling in lumbar MNs could enhance excitability through NMDAR-dependent mechanisms, a hypothesis that warrants further testing.

### BDNF alters transcripts encoding GABAA and glycine receptors in lumbar motoneurons

SCT followed by PBS injection decreased mRNA levels for most examined GABAA receptor subunits, glycine receptor subunits, and the K^+^/Cl^−^ cotransporter 2 KCC2, the latter consistent with prior reports [11, 18, 41, 42]. These cell-specific effects in isolated MNs were also detected in whole-segment analysis (L1-L2 and L3-L6) indicating a general downregulation of inhibitory receptor subunits in the interneuronal network. BDNF overexpression further reduced transcripts for *Gabrb3*,* Gabrg2*, with the strongest effect on *Glra1*,* and Glrb* in GL MNs, and *Gabra2* and *Glra1* in TA MNs. KCC2 transcript levels remained depressed in both MN populations despite BDNF overexpression, indicating persistent transporter deficiency regardless of TrkB activation. These findings are important because they argue against a simple model in which BDNF restores all components of inhibitory neurotransmission toward the uninjured state. Only the increase in *Gabra1* subunit levels in the whole lumbar segment conformed to this potential trend.

The observed mRNA changes do not establish whether the total number of GABAA or glycine receptors is reduced. They may instead reflect altered availability of selected subunits for receptor assembly and could affect the relative abundance, synaptic localization, or functional properties of specific receptor subtypes. BDNF/TrkB signaling can bidirectionally regulate KCC2 via distinct intracellular pathways [43], but its effects after SCI remain unclear. Reduced KCC2 contributes to hyperreflexia and spasticity in chronic SCI [18]. Activity-based therapies, such as treadmill training, alleviate these symptoms by increasing endogenous BDNF, which in these conditions promotes KCC2 insertion into the plasma membrane of lumbar MNs [44–46]. Elevated KCC2 restores Cl^−^ homeostasis, reduces MN hyperexcitability, and mitigates spasticity; blocking BDNF signaling prevents this effect. Intrathecal BDNF administration 15 days post-SCI similarly increases plasmalemmal KCC2, suggesting exogenous BDNF can attenuate spasticity after hyperreflexia develops [18].

**Fig. 7:**
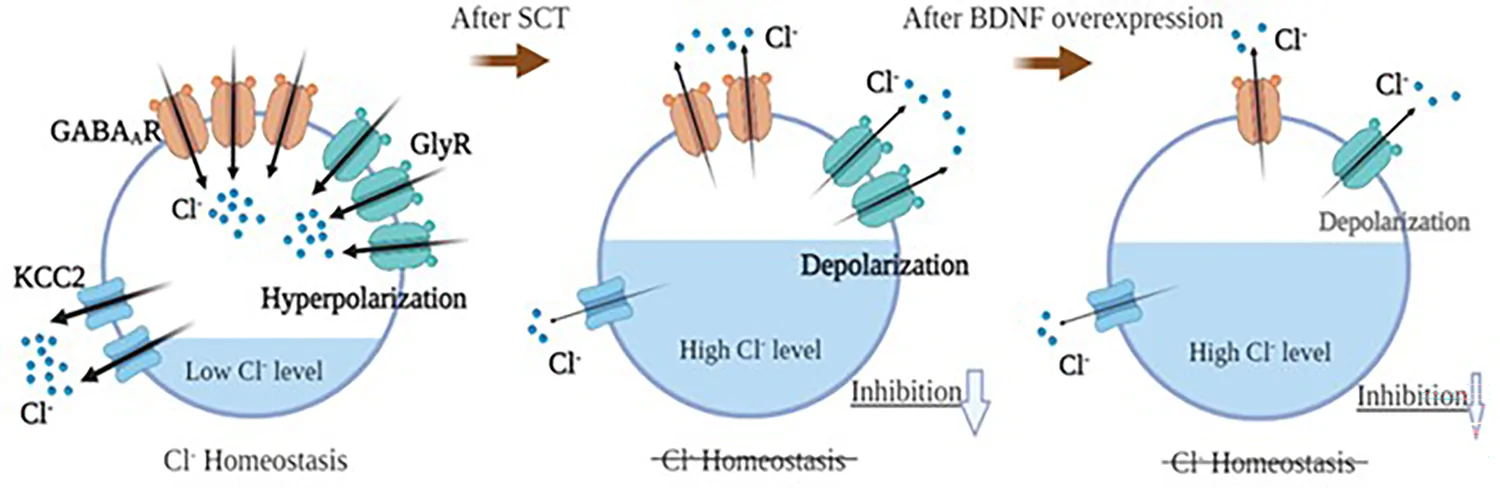
Proposed effects of SCT and BDNF overexpression on Cl⁻ homeostasis in motoneurons (MNs). KCC2 is the primary Cl⁻ extruder in mature neurons and maintains low intracellular Cl⁻ concentrations, supporting inhibitory GABAAR- and GlyR-mediated signaling. After spinal cord transection (SCT), reduced KCC2 expression may result in elevated intracellular Cl⁻ levels, and shift in GABAergic and glycinergic signaling toward depolarization. In the present study, BDNF overexpression did not restore KCC2 transcript levels, suggesting that altered Cl⁻ homeostasis may persist. Under these conditions, GABAAR and GlyR activation may become less inhibitory or even depolarizing. The f urther reduction in GABAAR and GlyR transcript levels following BDNF overexpression observed in this study may represent an adaptive response to altered ionic homeostasis and synaptic signaling. However, the present data do not establish whether these transcriptional changes affect total receptor abundance, receptor subtype composition, or receptor function

Contrary to our initial hypothesis based on the previous observations, enhanced BDNF signaling did not restore expression of receptor subunits and associated transporters mediating inhibitory neurotransmission in MNs. SCT-induced KCC2 downregulation likely leads to intracellular Cl^−^ accumulation in GL and TA MNs, shifting GABAAR and GlyR responses from inhibitory to depolarizing. Persistent KCC2 deficiency, combined with further GABAR and in particular GlyR transcript loss, suggests continued Cl^−^ dysregulation. Under these conditions, GABAAR and GlyR activation may paradoxically provide excitatory drive, representing a form of functional disinhibition that supports MN excitability- potentially a maladaptive mechanism of partial recovery (Fig. 7).

Our previous work provides additional context: prolonged BDNF administration (6–7 weeks) reduced KCC2 expression and protein level caudal to the lesion while increasing levels of GABA and the GABA-synthetic enzymes GAD65 and GAD67 [11]. This molecular profile coincided with hyperreflexia, consistent with high intracellular Cl⁻ levels driving GABA/GlyR-mediated Cl⁻ efflux, membrane depolarization, and increased Ca²⁺ influx via voltage-gated Ca²⁺ channels and NMDARs—collectively enhancing neuronal excitability [43]. Together, these findings raise a key question: What determines whether BDNF upregulates or downregulates KCC2 expression after SCI? The most plausible factor is BDNF concentration rather than its source. Physical training may promote physiologically appropr te BDNF synthesis and release due to spinal circuit plasticity, thereby alleviating SCI symptoms. In contrast, BDNF levels resulting from exogenous delivery of *bdnf* transgene is difficult to regulate and may exceed endogenous needs.

BDNF has been reported to downregulate KCC2 mRNA in non-axotomized corticospinal neurons but upregulate it in axotomized neurons, indicating that axotomy status modulates BDNF’s effects [47]. In our model lumbar MNs are not directly axotomized, although fiber retraction in response to altered connectivity cannot be excluded. Consistent with previous studies, neuronal loss was largely confined to the lesion site, whereas lumbar MNs remained structurally intact [48–50], as confirmed by our measurements of MN size in SCT-PBS and SCT-BDNF groups (Supplementary Fig. 1).

### Selectivity of BDNF-induced upregulation of serotoninergic 5-HT2A receptors

Here, we demonstrated that BDNF overexpression significantly upregulated 5-HT2A receptor (*Htr2a*) transcripts exceeding control levels - particularly in GL MNs - while α1-adrenergic receptor (ARα1A) expression remained unchanged. Low and inconsistent detection *of Htr2b* and *Htr2c* transcripts prevented reliable quantitative analysis. Therefore, the present study provides evidence for a strong effect of BDNF on *Htr2a* transcripts, but does not establish whether BDNF has similar effects on other 5-HT receptor subtypes. This finding is consistent with the possibility that BDNF/TrKB signaling enhances 5-HT2A-related serotonergic modulation of MN excitability after SCT, potentially supporting locomotor recovery after SCT. Moreover, BDNF may further modulate MN excitability not only via synaptic mechanisms but also by regulating Na⁺, Ca²⁺, and K⁺ channel gating [51, 52]. BDNF overexpression did not significantly affect *Cav1.3 or KCa1.1* transcript levels. However, it normalized the SCT-induced upregulation of *Nav1.1*—a key voltage-gated Na⁺ channel in GL and TA MNs. This selective enhancement of excitatory serotonergic signaling, together with normalization of Nav1.1 expression in GL and TA MNs, suggests that BDNF/TrkB signaling coordinately modulates synaptic and intrinsic excitability to support locomotor recovery after SCT.

Descending serotonergic (5-HT) and noradrenergic (NA) brainstem pathways critically regulate spinal circuit excitability and represent key therapeutic targets after SCI. Pharmacological activation of 5-HT and adrenergic receptors or transplantation of embryonic brainstem tissue, can restore coordinated hindlimb locomotion in adult animals [53–58]. At the cellular level, 5-HT2 and α1-adrenergic receptors modulate MN excitability, largely via facilitation of persistent inward currents (PICs) [59, 60]. BDNF has also been shown to promote 5-HT fiber sprouting and enhance serotonergic innervation of MNs [61–66], but whether BDNF directly regulates receptor expression after SCI remains unclear.

Notably, activation of 5-HT2A receptors has also been shown to restore Cl⁻ homeostasis and reduce spasticity by increasing KCC2 membrane expression in lumbar MNs and dorsal horn neurons [67, 68]. Although KCC2 transcript levels were not altered by BDNF overexpression in our study, we cannot exclude the possibility of post-transcriptional regulation or altered membrane localization of the KCC2 protein. Further studies are required to determine whether BDNF affects the protein expression or subcellular distribution of these molecules.

### Motoneuron subtype-specific responses and the relevance of proteomic profiling

A growing body of evidence, including this study, indicates that MNs innervating distinct muscle groups possess unique molecular and physiological properties. However, comprehensive proteomic characterization of target-defined MN populations remains technically challenging, largely because proteins cannot be amplified and high-throughput protein analyses in individual MNs are still limited.

Recent proteomic profiling of laser-microdissected, retrogradely labeled MNs [69] showed that Sol, a postural analogue of the GL, and TA MNs share most of their detected proteins with approximately 1200 identified and differing only in 38. Sol MNs showed higher expression of proteins associated with neuroprotection and cellular homeostasis, including Cst3, Picalm, Lnpk, Hagh, and Ckap4, whereas TA MNs were enriched in cytoskeletal regulators and LDHA, consistent with distinct metabolic and structural profiles.

Following spinal cord transection, proteomic remodeling was more extensive in Sol than in TA MNs, with earlier and more pronounced acute changes than chronic ones. These changes involve distinct protein sets, suggesting subtype-specific differences in vulnerability and adaptive capacity [69]. This pattern is consistent with the present findings, in which GL and TA MNs differed in both the magnitude and direction of their molecular responses to SCT and BDNF. Together, these observations support the concept that the effects of spinal cord injury and BDNF are shaped, at least in part, by the intrinsic molecular properties of individual MN subtypes. Target-defined analyses may therefore provide information that would be obscured when MN populations are considered collectively.

### Considerations on sex-dependent changes

To maintain continuity with our previous studies using the same complete spinal cord transection model, AAV-BDNF treatment, behavioral, and molecular analyses male rats were used in the current investigation. In light of several recent studies in humans and animals that demonstrate sex differences in motoneuron firing behavior, neurological impairment, physical performance and expression of genes related to ion channel function [70–73], the use of male rats only is a limitation of the study. Indeed, sex may affect functional, inflammatory, and molecular responses after SCI, but the direction and magnitude of these effects vary with the injury type, strain, time point after injury, spinal cord level under analysis, and outcome measure [74–80]. Gonadal hormones may contribute to these differences, as ovarian hormone status influenced respiratory recovery after cervical hemisection, and sex hormones affected acute glutamate responses after SCI [78, 79]. Recent findings underscore the importance of considering sex as a biological variable in regulation of 5HTR2a and 5HTR2c receptors both in the lumbar and sacral spinal cord [71], but the effect of sex onTrkB receptor expression profile in motor neurons and the interneuronal network of both male and female subjects remains to be characterized. Because sex-dependent regulation of TrkB, Htr2a, Adra1a, and other identified targets in lumbar motoneurons after complete SCI remains insufficiently characterized, we cannot reliably predict whether females would show greater or lesser BDNF-related effects. This should be addressed in future studies including both sexes.

## Conclusions

Short-term BDNF overexpression in lumbar spinal cord segments significantly improves locomotor performance in spinalized rats and preserves trophic signaling within spinal circuitry and MNs. Under these conditions, BDNF/TrkB signaling induces multidirectional changes in receptor and ion channel expression in lumbar MNs. Notably, differences in the magnitude and direction of these changes between GL and TA MNs suggest molecular distinctions between extensor and flexor signaling machinery that warrant further study.

This pattern of receptor and channel modulation indicates a shift toward disinhibition and enhanced neuromodulatory drive, which may contribute to elevated excitability in both extensor and flexor MNs. The net effect of BDNF on MN function turned out to be complex, reflecting a balance between adaptive excitatory enhancement and potential maladaptive disinhibition, and warrants further mechanistic investigation.

## Supplementary Information

Below is the link to the electronic supplementary material.

Supplementary Material 1

## Data Availability

Data supporting the findings of this study are available in the Supplementary Information or from the corresponding author upon request.
