# Supplementary material for: AAV-BDNF modulates 5HT2A receptor, glycine receptor subunits, and Nav1.1 channel gene expression in lumbar motoneurons after spinal cord injury
Source: Mol Biol Rep. 2026 Nov 1;53(1):1872. doi: 10.1007/s11033-026-12984-4 (PMC13724633; doi:10.1007/s11033-026-12984-4)
Supplement: Supplementary file 1 [file 11033_2026_12984_MOESM1_ESM.pdf]

Supplementary Materials

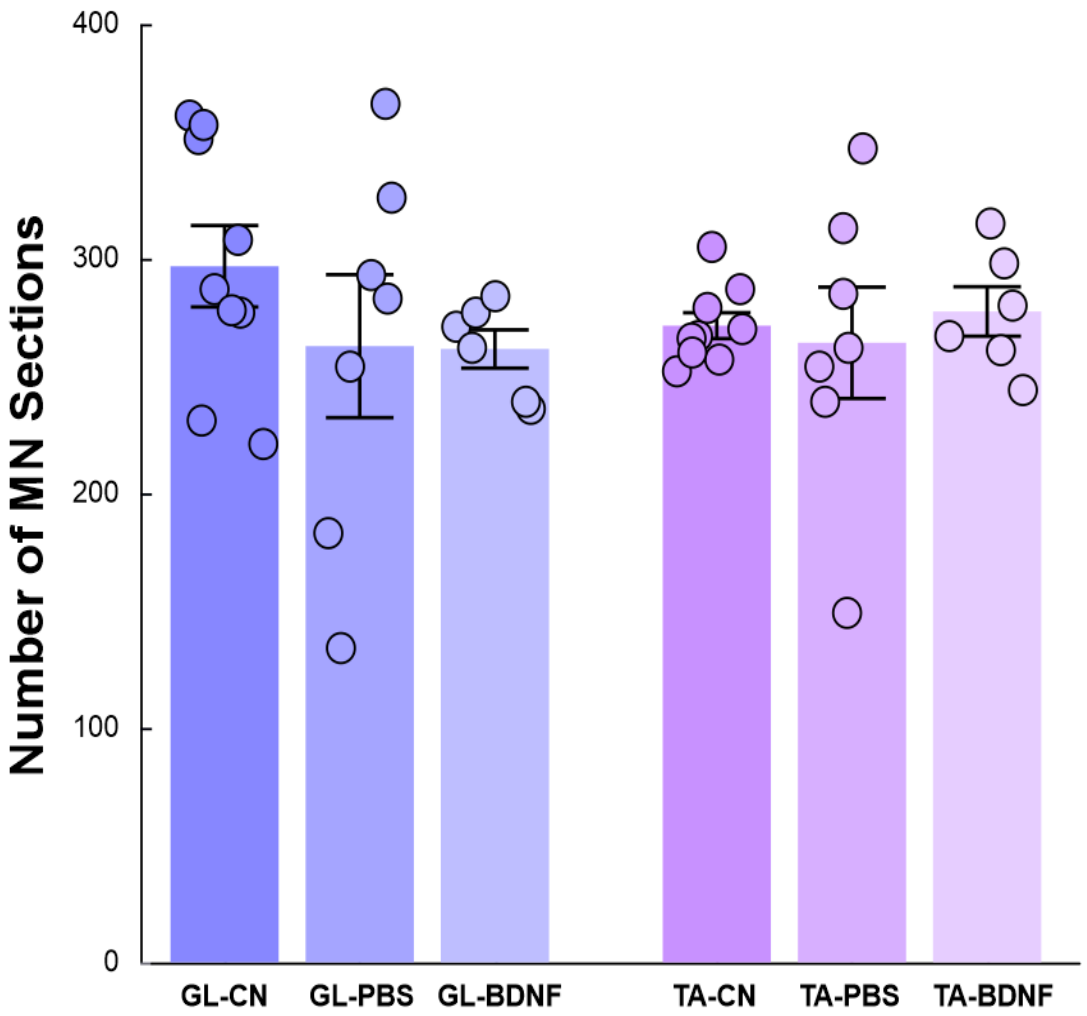

Fig S1. The number of GL and TA MNs isolated from the CN (N=9), SCT-PBS (N=7) and SCT-BDNF (N=6) groups of rats. Data are presented as mean +/- SEM. Differences between groups are non-significant (One-way ANOVA with Tukey's post-hoc test).

Table S1. List of used probes and primers

Gene symbol	Name of coded protein	Accession number	Roche UPL No.	Forward primer (5'–3')	Reverse Primer (5'–3')	Amplicon length/nt
Gria1	AMPAR GluA1	NM_031608.1	69	tcccttgaccataaccttgg	actcatgaatggcttggagaa	61
Gria2	AMPAR GluA2	NM_017261.2; NM_001083811.1	67	ggaggtgattccaaggaaaag	cccccgacaaggatgtaga	77
Gria3	AMPAR GluA3	NM_032990.2; NM_001112742.1	94	ccaagaatgcaccactgaag	ctgcgggagacatccact	109
Grin1	NMDAR GluN1	NM_001270602.1	95	cctacacagctggcttctacag	cgaaggaaactcaggtggat	94
Grin2a	NMDAR GluN2A	NM_012573.3	94	cgtcatggtctccaggagtaa	cactgaagggttcgaggaaa	62
Grin2b	NMDAR GluN2B	NM_012574.1	106	tcctgcagctgtttggagat	gctgctcatcacctcattctt	95
Gabra2	$GABA_AR$ α2	NM_001135779.2	94	gacagacttctggatggttacga	ggtcacatagatgttggtggag	87
Gabra3	$GABA_AR$ α3	NM_017069.3	129	cctacttgccatgtatcatgactg	ggtcatggtgagaacagtggt	113
Gabrb3	$GABA_AR$ β3	NM_017065.1	80	atcgagctcccacagttctc	tcaatgagagtcgagggtagg	91
Gabrg2	$GABA_AR$ γ2	NM_183327.1	75	ttgaagtgggagacacaaggt	cggacataaccacatagtcacc	111
Glra1	GlyR α1	NM_013133.1	69	ccggcaacacaaggaact	tctccaccctcatcatcctt	69
Glrb	GlyR β	NM_053296.1	29	ggatctattcaagagacaacaatgg	gcttgagtctggggtcgtt	79
Slc12a4	KCC1	NM_019229.2	101	acagagcgggatcgagag	ctctggtccatgtcatctgg	130
Slc12a5	KCC2	NM_134363.1	75	ttctggacaaccaccctca	ggcagaagcccttcacct	84
Slc12a6	KCC3	NM_001109630.1	124	gcgtcttacctgggtagtgg	tggagatggcagttaacattgta	95
Scn1a	Nav1.1	NM_030875.2	67	agaaccaggccacattggag	gttctgatgctgttgctgcc	120
Scn8a	Nav1.6	NM_019266.3	67	agagcaaaaagaggccgagt	tcttcaatggcgtcttccga	120
Scn9a	Nav1.7	NM_133289.2	129	ggtcatggtgattgggaacc	ttgtttgcatcggtgtcttcc	111
Cacna1b	Cav2.2	NM_001195199.1 NM_147141.1	73	cggcactggagatcaagc	gcagattagcccacacagaag	96
Cacna1d	Cav1.3	NM_001389225.2	69	cccttatccaccctgcgatg	agattcttcgaggacgggga	98
Kcnn2	SK2 (K_{Ca}2.2)	NM_001309404.1; NM_019314.2	113	gcgttttgttatgaagactttaatga	gtgacatcctgttgatcatggta	132
Kcnn3	SK3 (K_{Ca}2.3)	NM_019315.2	69	ttcagggctcccaattca	atttagctggctgccttgc	72
Kcnma1	KCa1.1	NM_031828.1	56	cctcacacagccctttgc	gggtgaggatattgtcattgaag	95
Grm1a	mGluR1A	NM_017011.1	101	caccgaagaagatgaattgga	gaatcctcaggggtcagctt	69
Grm5	mGluR5	NM_017012.1	82	tccagcagcctagtcaacct	gattttccgttggagcttagg	71
Chrm2	M2R	NM_031016.2	64	ccaccttcagactgtcaacaatta	catggagaaaacacctatgatgag	76
Htr2a	5-HTR2A	NM_017254.1	69	agcactcgagccaaactagc	tggaagagcttttctgatgaca	71
Htr2b	5-HTR2B	NM_017250.2	129	cgcggtaataatccccacca	ccaccgccaaggacattaga	110
Htr2c	5-HTR2C	NM_012765.3	69	aagaaggtgttcgttggcct	ccgtttctcgtctagctgcc	106
Adra1a	NAα1A	NM_017191.2	50	gcgtctgggtgctttctttg	ggctcctcattgatctggca	100
Adra1d	NAα1D	NM_024483.2	129	tgtgctggttccccttcttc	agcgggttcacacagctatt	124
GAPDH	GAPDH	NM_017008.4	*	ctgcaccaccaactgcttag	tgatggcatggactgtgg	92

*sequence: tttggcatcgtg. Synthesized by Institute of Biochemistry and Biophysics, Polish Academy of Sciences, Warsaw, Poland

Table S2. List of probes used for RNAScope method.

Probe	cat. no	Target description
Rn-Ngfr	406351	Rattus norvegicus nerve growth factor receptor (Ngfr), mRNA
Rn-Ntrk2-C2	317531-C2	Rattus norvegicus neurotrophic tyrosine kinase receptor type 2 (Ntrk2) transcript variant 1 mRNA
Rn-BDNF	customer designed probe, by ACDBio	Brain-derived neurotrophic factor (bdnf), mRNA BC087634.1
Polr2A C1 - positive control<br>PPIB C2 - positive control<br>UBC C3 - positive control	320891	DNA-directed RNA polymerase II subunit RPB1<br>cyclophilin B<br>ubiquitin C
DapB (C1, C2, C3) – negative control	320871	DapB gene (enzyme from Bacillus subtilis)

Table S3. Primary and secondary antibodies for immunofluorescence

Target	Host	Cat. no	Dilution	Producer
C-Myc tag	Mouse	9B11	1:500	Cell Signaling Technology, Danvers, MA, USA
Brain-derived neurotrophic factor (BDNF)	Rabbit	ANT-010	1:200	Alomone Labs, Jerusalem, Israel
Anti-Mouse, conjugated with Alexa Fluor 488	Donkey	A21202	1:500	Invitrogen, Carlsbad, CA, USA
Anti-Rabbit, conjugated with Alexa Fluor 568	Donkey	A-10042	1:500	Thermo Fisher Scientific, Waltham, MA, USA

Table S4. Behavioral parameters assessed during treadmill locomotion and open-field observation. Behavioral parameters were assessed during 1-min observations for both tests. For hindlimb movements, R and L indicate the right and left hindlimb, respectively. **Amplitude:** N, no movement; W, weak movement; S , small; M, medium movement; and L, large. **mBBB score:** modified Basso, Beattie, and Bresnahan locomotor rating scale, ranging from 0 (no observable hindlimb movement) to 21 (normal locomotion), assessing hindlimb movement, weight support, plantar stepping, and interlimb coordination. **Open-field score:** frequency of hindpaw movements, scored as 0, no movement; 1, low-frequency movement; 2, movements with a frequency comparable to that of the forelimbs and 3, movements with a frequency exceeding that of the forelimbs. **Symmetry:** S, symmetric hindlimb activity (similar R/L activity); A, asymmetric hindlimb activity.

		Treadmill locomotion (no tail stimulation)							Open Field		
Animal	Date of Post Surgery	Number of step-like movements (R or L)	Number of R-L alternations	Number of plantar steps R	Number of plantar steps L	Number of steps with body weight support	Ampli-tude	mBBB Score	Score [0-3]	Symmetry	Notes
**SCT-PBS**											
Bi1 (L view)	13	0	0	0	0	0	No	0	1	S	
Bi2 (R view)	13	1	1	0	0	0	No	0			
Bi3 (R view)	13	0	0	0	0	0	No	0			
Bi4 (L view)	13	74	0	0	5	0	W	2			
Bi5 (L view)	13	0	0	0	0	0	No	0			
Bi6 (L view)	13	0	0	0	0	0	No	0			
Bi7 (L view)	13	0	0	0	0	0	No	0			
Zc4 (R view)	13	0	0	0	0	0	No	0	1	S	
Zc5 (L view)	13	0	0	0	0	0	No	0	1	S	L hindlimb 2 movements
Zc6 (L view)	13	2	0	0	0	0	No	1	1	S	L hindlimb 1 movement
Zc7 (R view)	13	0	0	0	0	0	No	0	0	S	
Zc8 (R view)	13	0	0	0	0	0	No	0	2	S	R hindlimb 2 movements
Zc9 (L view)	13	0	0	0	0	0	No	0	0	S	
Zc10 (L view)	13	6	0	0	0	0	No	2	1	S	L hindlimb 2 movements
**SCT-BDNF**											
Bj1 (R view)	13	63	29	57	41	41	L	16	1	A (L on the top)	Only L is moving
	14	52	37	52	48	38	M/S	17			
Bj2 (R view)	13	59	28	55	41	43	W	16	2	S	Both R, L on plantar side, alternative movements, exceptionally good
	14	48	18	38	34	30	M-	15.5			
Bj3	13	52	17	30	35	23	S/M-	13	3	S / A (L on the top)	Occasional strong abduction of the L hindlimb
Bj4 (L view)	13	47	8	26	29	27	L	11,5	2	A (L on the top)	moments of plantar foot placement
	14	49	10	38	47	42	M	15.5			
Bj5 (L view)	13	42	3	16	13	6	W	11	3	A (L on the top)	L hindlimb active, lower or higher frequency than forelimb
	15	55	7	30	45	19	S	11.5			
Bj6	13	50	46	46	46	50	M/S	18	2	S / A (L on the top)	Occasional plantar steps with alternations
Bj7 (L view)	13	32	3	7	3	0	W	11	1	S	Occasional plantar foot placement
	16	57	24	46	46	29	M/S	16			
Zd1 (L view)	13	47	2	12	3	1	W	4.5	1	A (R on the top)	R hindlimb frequent abduction, scratch the ground
	16	51	18	24	37	27	W	15.5			
Zd2 (R view)	13	35	11	16	10	5	W	11	2	S	L abduction and scratch like movements; R- good
	14	70	36	64	68	52	L	16.5			
Zd3 (R view)	13	41	21	26	24	2	W	11	3	A (L on the top)	Successfully climbing on cage-box
	16	38	12	33	38	13	S	11.5			
Zd4 (L view)	13	61	8	11	50	7	W	11	3	S / A (L on the top)	Successfully climbing on cage-box, low frequency symmetric
	16	62	8	30	37	30	M	11			
Zd5 (L view)	13	43	17	28	39	12	W+	11.5	2	S	frequent body weight support, R leg less active
	15	43	18	33	36	24	S	15.5			
Zd7 (R view)	13	59	24	53	39	29	W	13	2	S	Successfully climbing on cage-box
	17	32	5	29	11	20	M/S	11.5			

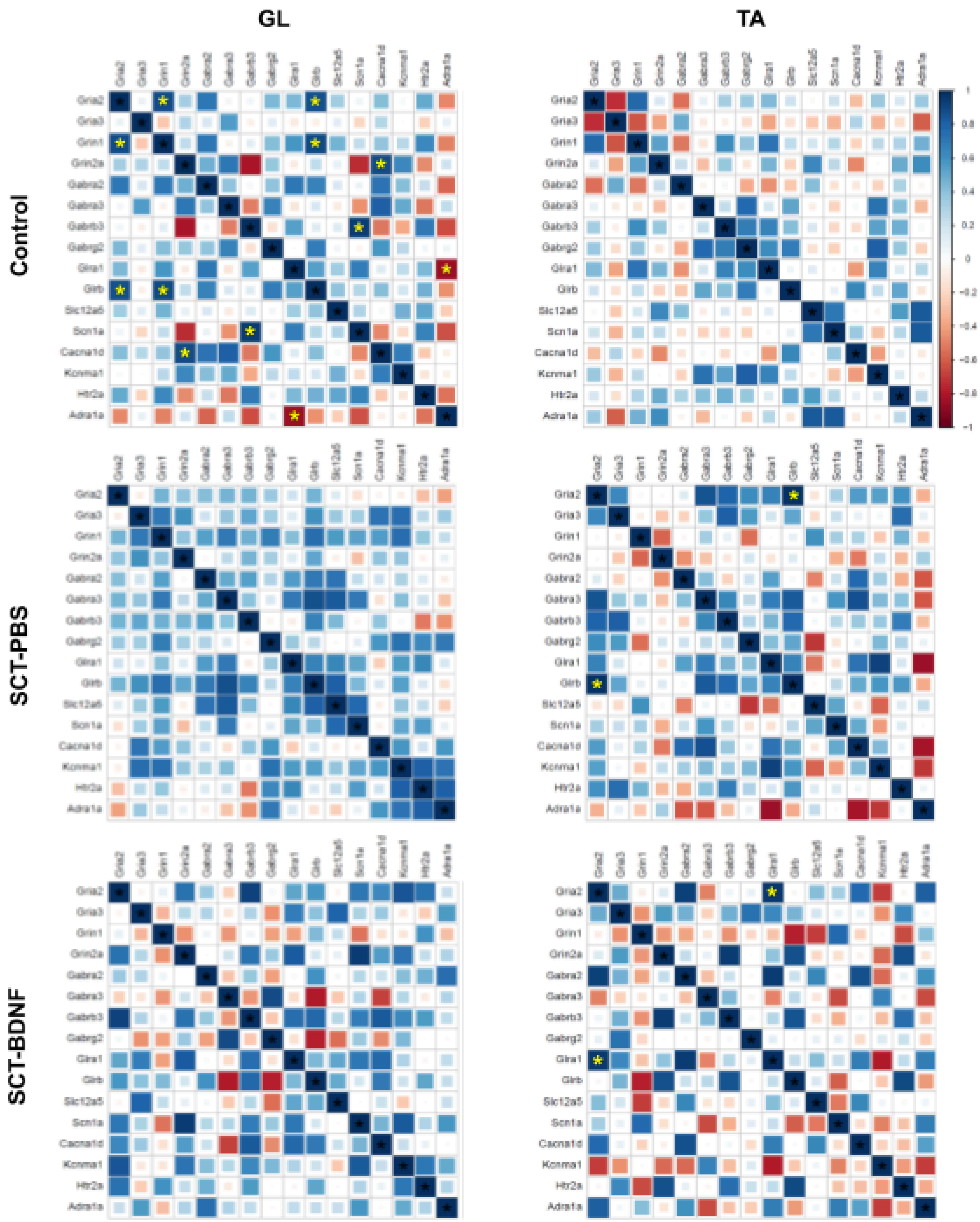

Fig S2. Spinal cord transection and BDNF overexpression alter gene co-expression networks in GL and TA motoneurons. Heatmaps show Spearman correlation coefficients among gene transcript levels in GL (left) and TA (right) motoneurons from Control, SCT-PBS, and SCT-BDNF groups. Asterisks indicate statistically significant correlations ($p < 0.05$). In GL MNs, SCT-PBS disrupted several correlations present in Control groups and introduced new clusters of positively correlated genes. In TA MNs, gene–gene correlations were generally weaker in ControlsN but became more structured following SCT and BDNF treatment, reflecting treatment-specific reorganization of gene co-expression patterns. The order of the genes along the X- and Y-axis of each correlogram are the same for comparison of the changes between groups. * Significant FDR corrected p-value in R.
